# Loss of Control as a Discriminating Factor Between Different Latent Classes of Disordered Gambling Severity

**DOI:** 10.1007/s10899-016-9592-z

**Published:** 2016-02-18

**Authors:** Richard J. E. James, Claire O’Malley, Richard J. Tunney

**Affiliations:** 1 School of Psychology, University of Nottingham, University Park, Nottingham, NG7 2RD UK; 2 School of Psychology, University of Nottingham Malaysia Campus, Jalan Broga, 43500 Semenyih, Selangor Darul Ehsan Malaysia

**Keywords:** Problem gambling, Gambling prevalence, Latent class analysis, Assessment

## Abstract

Analyses of disordered gambling assessment data have indicated that commonly used screens appear to measure latent categories. This stands in contrast to the oft-held assumption that problem gambling is at the extreme of a continuum. To explore this further, we report a series of latent class analyses of a number of prevalent problem gambling assessments (PGSI, SOGS, DSM-IV Pathological Gambling based assessments) in nationally representative British surveys between 1999 and 2012, analysing data from nearly fifty thousand individuals. The analyses converged on a three class model in which the classes differed by problem gambling severity. This identified an initial class of gamblers showing minimal problems, a additional class predominantly endorsing indicators of preoccupation and loss chasing, and a third endorsing a range of disordered gambling criteria. However, there was considerable evidence to suggest that classes of intermediate and high severity disordered gamblers differed systematically in their responses to items related to loss of control, and not simply on the most ‘difficult’ items. It appeared that these differences were similar between assessments. An important exception to this was one set of DSM-IV criteria based analyses using a specific cutoff, which was also used in an analysis that identified an increase in UK problem gambling prevalence between 2007 and 2010. The results suggest that disordered gambling has a mixed latent structure, and that present assessments of problem gambling appear to converge on a broadly similar construct.

**Electronic supplementary material:**

The online version of this article (doi:10.1007/s10899-016-9592-z) contains supplementary material, which is available to authorized users.

## Introduction

One of the debates in defining disordered gambling is whether disordered gamblers form the extreme of a continuum of severity, or whether there are qualitative differences between disordered and non-disordered gamblers. Studies of disordered gambling using taxometric analysis have identified a qualitatively distinct latent class of gamblers showing very high problem severity (James et al. 2014; Kincaid et al. 2013). Widely supported models of gambling disorder, such as the Pathways Model (Blaszczynski and Nower 2002), hypothesize the presence of latent classes amongst problem and pathological gamblers (Blaszczynski 2000). Other studies utilising latent class analysis (LCA) to determine the number of discrete subtypes have demonstrated mixed findings. LCA studies of pathological gambling have consistently found three or four subtypes of gambler. Some studies have concluded that there are quantitative and qualitative differences between latent classes (Nower et al. 2013; Xian et al. 2008), and others have emphasized that the ordering of the subtypes are evidence for a dimension (Carragher and McWilliams 2011; McBride et al. 2010). Although arguing that the evidence was stronger for a dimension of severity, these haven’t excluded the possibility of qualitative differences amongst gambling subtypes (McBride et al. 2010). The latent classes were similar across studies, comprising one group displaying no/minimal symptoms, a group showing moderate probability of symptom endorsement, and a group that exceeded the DSM cutoff for Pathological Gambling. Other analyses of prominent gambling assessments support a continuum of severity (Miller et al. 2013; Strong and Kahler 2007), but these use analytic methods that already assume a latent dimension is being measured. To examine this further, this report describes the findings of seventeen LCAs across five different surveys of the British population over a fifteen year period, using four assessments measuring problem and pathological gambling constructs.

Between 1999 and 2012, five nationally representative British and English surveys included assessments of disordered gambling. Three of these (the British Gambling Prevalence Survey series (BGPS)) (Sproston et al. 2000; Wardle et al. 2007, 2011) surveyed gambling behaviours, attitudes and disordered gambling prevalence in the UK, and were conducted by the National Centre for Social Research. The initial BGPS (Sproston et al. 2000) assessed gambling in Britain following substantial changes in the gambling market (i.e. introduction of the National Lottery, scratchcards, internet gambling), and in anticipation of liberalized gambling legislation. The BGPS 2007 provided a baseline measurement of gambling in the UK prior to the implementation of the 2005 Gambling Act, and in light of changes since 1999. The BGPS 2010 intended to assess the impact of the Gambling Act introduced in September 2007. Measures of disordered gambling were included in three other surveys, commissioned by the Health and Social Care Information Centre or the Scottish Government; the Adult Psychiatric Morbidity Survey 2007 (APMS 2007) (McManus et al. 2009; Wardle et al. 2012), the Health Survey for England 2012 (HSE 2012) (Craig and Mindell 2013; Wardle and Seabury 2013), and the Scottish Health Survey (SHS 2012) (Rutherford et al. 2013a, b; Wardle 2013).

This period is one in which the potential for gambling-related harm increased following one of the two major phases of deregulation in the British gambling market, the other being the legalisation of off-course gambling in the mid to late 1960s (Orford 2010). During this period, electronic gaming machines (or FOBT’s) were legalised for use in high street bookmakers, online gambling emerged, and regulations on gambling advertisement were relaxed. It also covers a period in which the number of bookmakers increased considerably, following a decrease in the early to mid-1990s (Snowdon 2013). Analysis of BGPS 2007 and 2010 data showed a significant increase in problem gambling between 2007 and 2010 (Wardle et al. 2011) using a measurement derived from the DSM-IV Pathological Gambling criteria, albeit with strong caveats attached. Not least because the rate of disordered gambling is small, the observed increase prevalence amounts to less than twenty individuals.

The surveys included four assessments of disordered gambling. The first two were derived from the DSM-IV Pathological Gambling criteria (American Psychiatric Association 2000). In four of the surveys participants were given an adaption of the ten criteria, eliciting endorsement of a four-point scale of frequency (Fisher 1996), subsequently dichotomized as present or absent. For the first seven criteria, indicators were scored as present if endorsed at the two highest levels of frequency. For the final three items, responses other than ‘never’ was scored as present (Sproston et al. 2000). However, this differs from the logic of the DSM as individuals displaying disordered gambling behaviours might not be categorised as showing a specific symptom. Other analyses of BGPS data (McBride et al. 2010) have addressed this by re-dichotomizing the data on present/absence, present defined as a score greater than 0. In the APMS, respondents were asked to respond yes/no if they engaged in each of the ten criteria at any point over the previous 12 months.

In addition, the Problem Gambling Severity Index (PGSI) was included in four surveys (BGPS 2007/2010 and HSE/SHS 2012). The PGSI is the predominant contemporary population assessment of problem gambling (Williams et al. 2012). It is assumed to measure a continuum of harm (Ferris and Wynne 2001; Miller et al. 2013), but has been demonstrated to measure latent categories (James et al. 2014; Kincaid et al. 2013). The PGSI is partly derived from the DSM-IV Pathological Gambling criteria and the South Oaks Gambling Screen (SOGS) (Stinchfield 2002; Svetieva and Walker 2008), a pathological gambling assessment derived from the DSM-III criteria (Lesieur and Blume 1987), and administered in the BGPS 1999. The once popular SOGS has declined in use because it has been found to produce inflated pathological gambling estimates (Sproston et al. 2000; Stinchfield 2002). The questionnaire content, focusing on the financial consequences of gambling, has been criticised as not comprehensively measuring a pathological gambling model (Stinchfield 2002). However, the SOGS is still frequently used as a screen in experimental research. While it has been argued that these assessments might converge on the same construct (Svetieva and Walker 2008), this has not been directly tested. The PGSI and SOGS have not previously been analysed using LCA.

The aims of this study are fourfold. First, LCA’s of PGSI and DSM-IV data are warranted as both measure latent categories and might measure different constructs (James et al. 2014; Kincaid et al. 2013; Wardle et al. 2011). Second, this report aims to establish whether the latent structure of gambling disorder is consistent across time, as availability and accessibility are key components of many disordered gambling theories (Blaszczynski and Nower 2002), and the data covers a period where substantial changes occurred in the British gambling market. Also, LCAs comparing different DSM-IV assessments are useful to test whether screens that have elicited indicators in a different manner to the DSM retain a similar structure. Moreover, many of these assessments subtype gamblers (SOGS/PGSI), an approach taken by the DSM-5, or researchers often subtype sub-clinical gamblers (DSM-IV), and it is of interest to assess the validity of these distinctions.

## Methods

### Sample

The five surveys sampled 48,777 respondents. However, respondents were excluded if data was missing, or did not complete an assessment as they were under 16 or hadn’t gambled in the previous year, leaving 27,219 participants (see Table 1 for full details about the sample). The anonymised survey data for these analyses was downloaded from the UK Data Archive (National Centre for Social Research 2008, 2010, 2011; National Centre for Social Research & University College London. Department of Epidemiology and Public Health 2014; Scottish Centre for Social Research and NatCen Social Research & Survey Research Centre 2015). The data for each of the surveys was collected by interviewers employed by NatCen. The interviewers were briefed by the study lead researchers prior to data collection. They were given training on the questionnaire content, and instructed on the administration of the project and fieldwork protocol (Sproston et al. 2000; Wardle et al. 2011). After the sampling was carried out (see below), selected households were sent an advance letter informing them about the survey, and that they would be interviewed face to face for data collection (for the 2010 iteration the survey was administrated with computer assistance).

**Table 1:** Descriptive statistics for each of the problem gambling assessments, from each sample (weighted)

Sample	N	% >0 on screen	% lower PG threshold	% higher PG threshold	Cronbach’s α
BGPS 1999	7680 (5543—72 %)				
DSM—BGPS	5253	4.80 %	0.78 %	0.38 %	0.77
DSM—>0	5253	21.05 %	3.24 %	1.29 %	0.72
DSM—Polytomous	5253	21.05 %	N/A	N/A	0.78
SOGS	5010	13.25 %	13.25 %	1.22 %	0.79
BGPS 2007	9003 (6085—67.58 %)				
DSM—BGPS	5412	7.96 %	0.92 %	0.46 %	0.71
DSM—>0	5412	22.12 %	4.03 %	1.33 %	0.72
DSM—Polytomous	5412	22.12 %	N/A	N/A	0.77
PGSI	5486	10.63 %	2.97 %	0.80 %	0.9
APCS 2007	7393 (4826—65.76 %)				
DSM—yes/no	3628	5.79 %	1.19 %	0.55 %	0.81
BGPS 2010	7756 (5665—73.04 %)				
DSM—BGPS	5651	6.81 %	1.26 %	0.6 %	0.78
DSM—>0	5651	25.92 %	5.24 %	2.04 %	0.75
DSM—Polytomous	5651	25.92 %	N/A	N/A	0.81
PGSI	5657	11.05 %	3.45 %	1.01 %	0.9
HSE and SHS 2012	13,106 (7506—64.98 %)				
DSM—BGPS	6753	4.59 %	0.59 %	0.24 %	0.79
DSM—>0	6753	19.62 %	2.93 %	1.14 %	0.75
DSM—Polytomous	6753	19.62 %	N/A	N/A	0.81
PGSI	6787	7.16 %	2.11 %	0.47 %	0.91

The PGSI cutoffs reported here are 3+ and 8+ (Ferris and Wynne 2001). The DSM cutoffs reported are 3+, based on the BGPS report and 5, based on the cutoff for Pathological Gambling (American Psychiatric Association 2000; Sproston et al. 2000). For the SOGS, the cutoff’s are 1–4 for ‘gambling problems’, 5+ for ‘probable pathological gambler’(Lesieur and Blume 1987)

The BGPS 1999 (Sproston et al. 2000) was a nationally representative survey of the British general population aged 16 or older. The survey sampled 7680 respondents from a random sample of 7000 UK postcodes (response rate = 65 %). The survey found that 72 % of the sample had gambled in the previous year. 5289 respondents, 95 % of past-year gamblers, fully completed at least one pathological gambling assessment (DSM-IV ordinal response, SOGS).

The BGPS 2007 (Wardle et al. 2007) sampled 9003 respondents from a stratified sample of 10,114 addresses taken from the UK Postcode Address File, with the sample stratified by Government Office Region, socio-economic status and ethnicity. The response rate was 52 % and 68.4 % of the population gambled in the previous year. In total 5635 respondents, 91.4 % of past-year gamblers, fully completed a problem (PGSI) or pathological (DSM-IV ordinal) gambling assessment.

The APMS 2007 (McManus et al. 2009; Wardle et al. 2009) was the third in a series of surveys investigating psychiatric disorders, conducted by NatCen in collaboration with the University of Leicester, on behalf of HSCIC. This survey sampled 7403 respondents from a representative English sample (response rate of 57 %). Households were randomly selected from a stratified sample of English postcodes. One person was randomly selected from each household to complete the survey. The prevalence of past year gambling was 65.9 %, and a total of 3568 respondents (73 % of gamblers) fully completed the DSM-IV Pathological Gambling criteria based (yes/no) assessment included in this survey.

The BGPS 2010 (Wardle et al. 2011) was a nationally representative sample of British households, conducted by NatCen on behalf of the Gambling Commission. A total of 7756 respondents completed the survey, with households being randomly sampled from a stratified sample (stratified by the same variables as the BGPS 2007) of 391 postcode sectors. The response rate was 47 %, 73 % gambled over the previous year, and 5706 respondents fully completed a problem (PGSI) or pathological (DSM-IV ordinal) gambling assessment.

A module of gambling questions was included in the HSE (Craig and Mindell 2013; Wardle and Seabury 2013) and the SHS (Rutherford et al. 2013a; Wardle 2013) 2012. This data was drawn from a combined and reweighted sample based on a secondary analysis conducted by NatCen (Wardle et al. 2014). In total 16,935 respondents (10,333 English, 6602 Scottish) completed the health surveys. In total 13,106 were asked about their recent gambling behaviour (8291 England, 4815 Scotland). 65 % had gambled in the previous 12 months. Of those, 7021 (4290 England, 2731 Scotland) fully completed a problem (PGSI) or pathological (DSM-IV ordinal) gambling assessment.

### Measures

Gambling disorder was assessed via four methods: two DSM-IV Pathological Gambling based screens, PGSI and SOGS. The DSM measure included in the BGPS elicited each criterion on a 4-point scale of frequency. We conducted LCA on this data in three formats (ordinal data, dichotomised using BGPS approach, dichotomised based on present/absent). The assessments are reported in full in Table S1.

All five surveys included a measure based on the DSM-IV Pathological Gambling criteria, which assesses the presence of ten symptoms, classified as present/absent based on past year prevalence. Respondents endorsing five or more symptoms were classified as a pathological gambler. The DSM-5 (American Psychiatric Association 2013) uses a cutoff of four for Gambling Disorder. The BGPS reports use a cutoff of three to measure sub-clinical PG (Orford et al. 2010). For four of the surveys (BGPS series, HSE/SHS 2012), a questionnaire designed by Fisher (1996), and validated prior to the administration of the BGPS 1999 (Sproston et al. 2000) was used, with items probing each criteria elicited on a four point scale of frequency. In the APMS 2007, respondents were asked yes/no if they engaged in the behaviour covered by each criteria.

The PGSI (Ferris and Wynne 2001) is a nine-item assessment of problem gambling, designed to measure a continuum of gambling harm, elicited on a four-point scale of past-year frequency. The PGSI was administered in the BGPS 2007, 2010 and HSE/SHS 2012 surveys. The PGSI is a comparatively superior assessment of problem gambling (McMillen and Wenzel 2006). The PGSI discriminates four levels of problem gambling severity; non problem gambler (0), low risk problem gambler (1–2), moderate risk problem gambler (3–7) and problem gambler (8+). However, the validity of the intermediate interpretive categories used in the measure has been questioned (Currie et al. 2013), and the appropriate cutoff score to determine which individuals are of interest (Walker and Blaszczynski 2011). Several items in the PGSI are derived from the DSM-IV Pathological Gambling criteria or the SOGS.

The SOGS is a 16-item questionnaire derived from the DSM-III Pathological Gambling criteria (Stinchfield 2002). SOGS scores can range from 0 to 20,^1^ probing numerous problem and pathological gambling behaviours, including loss-chasing, guilt from gambling, lying to, receiving criticism from, and arguing with people close to the respondent about their gambling. Half of the SOGS items pertain to borrowing money, selling items or taking loans/credit out to fund gambling. A score of 0 is classified as non-pathological gambling, between 1 and 4 as having some problems with gambling, and 5 or more as a probable pathological gambler. The SOGS was adapted for the BGPS 1999 to measure past year, rather than lifetime, pathological gambling (Sproston et al. 2000).

### Analytic Procedure

LCA was conducted on each disordered gambling screen from each survey, on each case where a completed assessment was present. The indicators included for each analysis were the individual questions from each screen. The analysis was conducted using MPlus 6.1.3 (Muthén and Muthén 1998–2011). One through six-class models were compared in each analysis. LCAs were adjusted for survey weight (which differed depending on the sample),^2^ clustering and stratification.^3^ Interpretation of competing latent class models was conducted using multiple indices of fit. A number of different indices of fit can be used to determine which latent class model is appropriate, as there is no objective method for determining a latent class model to adopt. These include the Akaike Information Criterion (AIC) (Akaike 1974), Bayesian Information Criterion (BIC) (Schwarz 1978), Sample-Size Adjusted BIC (SSABIC) (Sclove 1987), and adjusted likelihood ratio tests (LRT) (Lo et al. 2001). Lower information criteria indicate superior model fit. LRT’s test the likelihood that a *k*-class model is a better fit of the data compared to a *k* − 1 class model, and reports a *p* value. If the *p* value is not significant, it is not possible to reject the *k* − 1 class model (Muthén and Muthén 1998–2011). Greatest weight was given to BIC, as previous studies have indicated its effectiveness at discriminating between latent class models (Nylund et al. 2007). Some methods appear to be more efficacious than others, but these appear to interact with a number of factors, with sample size for example an important consideration in determining whether to place greater weight on BIC or AIC. A number of studies have supported the use of a bootstrap variant of the likelihood ratio test (Nylund et al. 2007), but this test cannot be calculated for latent class models that account for complex sampling methods, such as the latent class models described in this report. The proportion of cases assigned to each latent class was determined based on the estimated model. In addition, the probability of endorsement for each level of each indicator for each latent class was calculated, and the posterior probability that each case assigned to a specified latent class.

Local independence was initially tested by assessing the Chi square for the overall model. It has previously been suggested that this is the most appropriate test for violations of local independence (Asparouhov 2015). A significant result indicates residual dependence between indicators at the level of the latent class or classes. In these situations looking at the bivariate residuals is advised to direct where local independence should be relaxed.

## Results

In the results that follow we report a number of observations. The first section gives an overview of the output of the LCAs, identifying the pattern of the similarities across analyses, and the profile of gamblers that fall into the different latent classes. The following section covers several indices of fit that may be used to justify selecting a specific latent class model. After this a high level overview of the level of consistency between different LCAs is reported. In light of these findings, consideration is then given to differences in demographic profiles and gambling behaviours, as research has tended to identify that the most severely disordered gamblers form different demographic profiles and engage in a range of addictive behaviours such as drinking and smoking. This is also informative to the literature concerning whether specific types of gambling game are linked with problematic behaviours. Finally, more detailed results are provided for each of the measurements used in the surveys covered in this analysis. For the DSM-IV based measure used in the gambling prevalence and health surveys, this contrasts between different methods of elicitation identified by the study authors and in the literature.

**Fig. 1:**
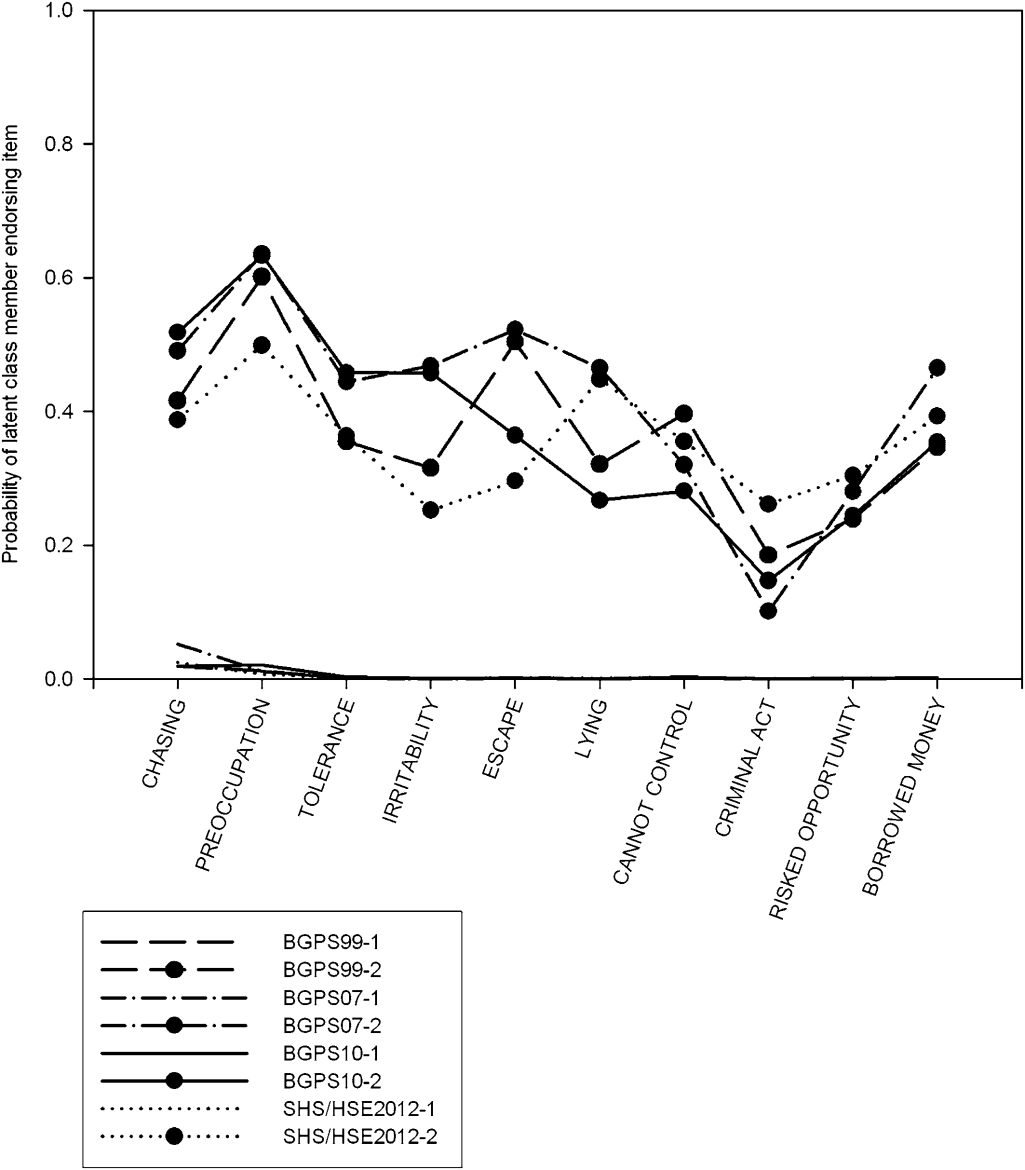
Plot of response probabilities for each item of the DSM-IV Pathological Gambling derived assessment, for two class solutions using the scoring method adopted in the BGPS reports (items rated from 0 to 3 by respondent, scored as present on items 1–7 if >1, on items 8–10 if >0). Latent classes are sorted by severity (lowest first)

**Fig. 2:**
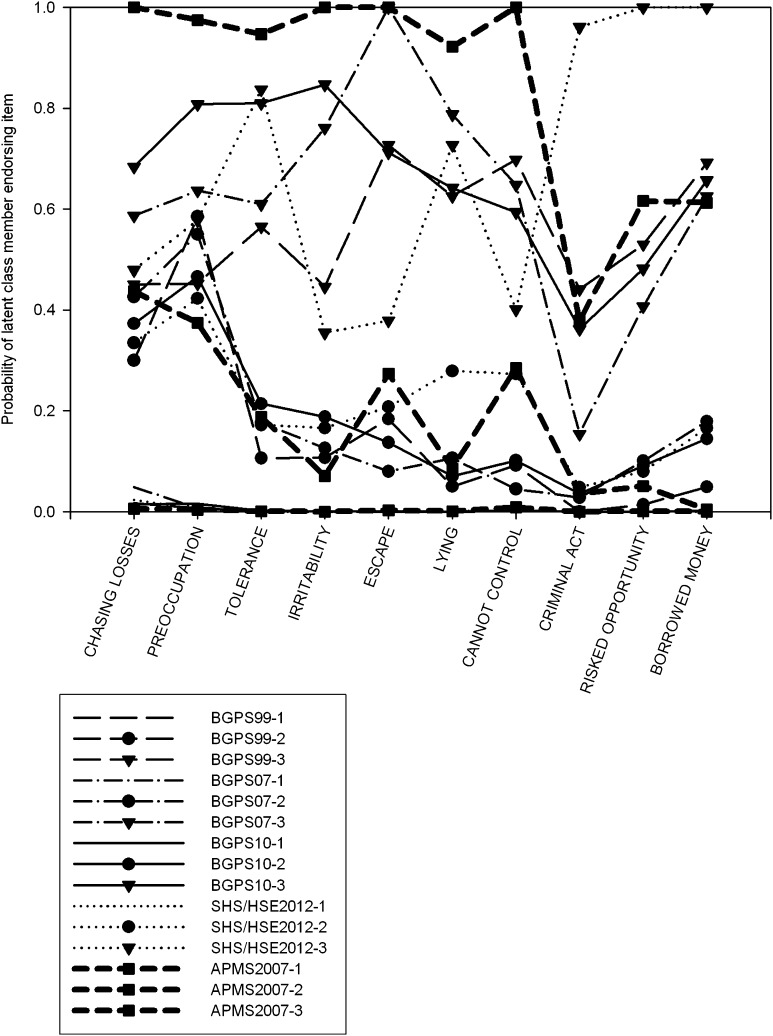
Plot of response probabilities for each item of the DSM-IV Pathological Gambling derived assessment, for three class solutions using the scoring method adopted in the BGPS reports (items rated from 0 to 3 by respondent, scored as present on items 1–7 if >1, on items 8–10 if >0). Latent classes are sorted by severity (lowest first)

**Fig. 3:**
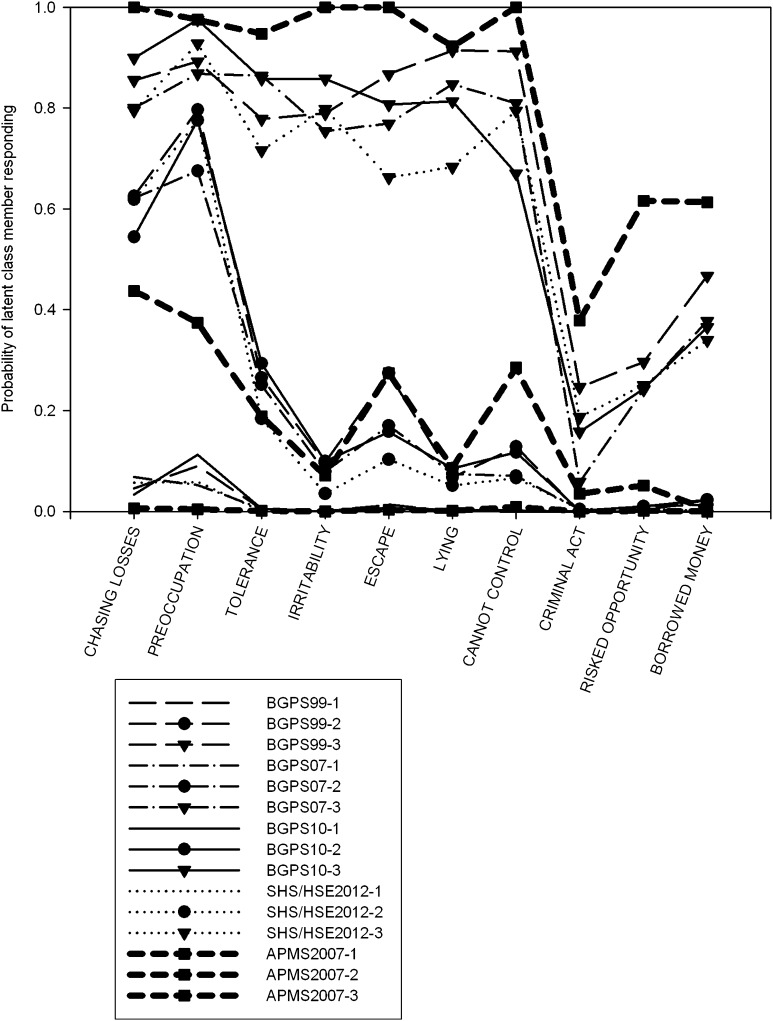
Plot of response probabilities for each item of the DSM-IV Pathological Gambling derived assessment for three class solutions, with symptoms scored as present if a response other than ‘Never’ (or 0) was given. Latent classes are sorted by severity (lowest first)

**Fig. 4:**
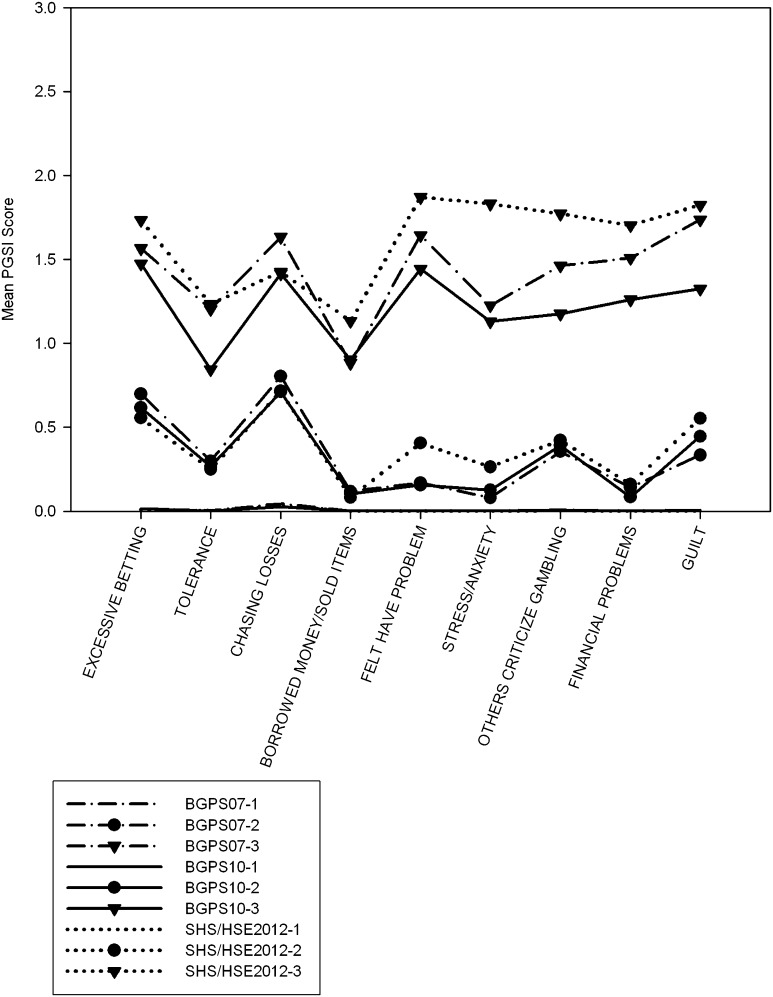
Plot of mean scores for each item of the Problem Gambling Severity Index items, three latent class solutions. Latent classes are sorted by severity (lowest first)

Fifteen of seventeen LCAs supported a three-class model. A summary table of 2–4 class models is reported in Table 2 (full results are reported in Tables S2–S6), which reports the full details of indices of fit for the estimated models. The three classes were consistent across measures: one class comprising 90–95 % of the sample showed minimal probability of endorsing any disordered gambling indicator, a second had a high probability of endorsing preoccupation and loss-chasing indicators, and a third had a high probability of endorsing many indicators. Response probabilities, standard errors for these, and the proportion of individuals assigned to each class for the estimated models are reported in Tables S7–S15. These indicated that differences between the second and third classes were primarily on items related to loss of control (Figs. 1, 2, 3, 4). These showed the largest separation between the two groups. The highest severity items (committing crimes, risked important opportunity, asked others for help with gambling financial difficulties) showed large differences but only had a moderate probability of endorsement by the third class. Means are reported for ordinal measures using most likely latent class membership (Tables S14, S15). An examination of the score distributions for each class based on most likely class membership indicated very little overlap in scores, suggesting that problem gambling falls along a dimension of severity (Tables S16–S20). AIC indices indicated a minimum of six latent classes on each LCA, although this appears to be because AIC over fits latent class models with many cases (Nylund et al. 2007). Classification accuracy was generally very high across measures, and did not appear to systematically differ between classes. In addition, information about demographic and game prevalence information for each latent class is reported in Tables S21 through S26.

**Table 2:** Summary indices from latent class analyses

	BIC	LMR-LRT *p*		BIC	LMR-LRT *p*
DSM-IV—BGPS Scoring			DSM-IV—Polytomous		
BGPS 1999			BGPS 1999		
2-class	3879.849	<.0001	2-class	14,237.622	<.0001
3-class	**3871.617**	.0104	3-class	**14,056.334**	**.0038**
4-class	3915.116	**.0327**	4-class	14,204.715	.7766
BGPS 2007			BGPS 2007		
2-class	5295.76	**<.0001**	2-class	16,724.245	<.0001
3-class	**5293.249**	0.1013	3-class	**16,363.372**	**.0278**
4-class	5351.032	0.2883	4-class	16,434.601	.762
BGPS 2010			BGPS 2010		
2-class	5845.435	**<.0001**	2-class	19,600.095	<.0001
3-class	**5819.839**	0.1708	3-class	**19,124.905**	**.0026**
4-class	5863.602	0.5028	4-class	19,182.884	.7866
SHS/HSE 2012			SHS/HSE 2012		
2-class	4652.381	**<.0001**	2-class	17,245.979	**<.001**
3-class	**4642.852**	0.2627	3-class	**16,880.958**	0.7259
4-class	4698.9	0.502	4-class	16,918.966	0.7699
DSM-IV—>0 Scoring			PGSI		
BGPS 1999			BGPS 2007		
2-class	11,592.606	<.0001	2-class	9977.427	**.0032**
3-class	**11,328.402**	**<.0001**	3-class	**9678.144**	.1543
4-class	11,344.377	0.1449	4-class	9683.177	.828
BGPS 2007			BGPS 2010		
2-class	13,287.834	<.0001	2-class	11,339.296	<.0001
3-class	**12,953.825**	**.0004**	3-class	10,988.334	**.0222**
4-class	12,971.168	.087	4-class	**10,986.805**	.7769
BGPS 2010			SHS/HSE 2012		
2-class	15,560.675	<.0001	2-class	8998.434	**<.0001**
3-class	**15,086.033**	**.0007**	3-class	**8837.362**	.0662
4-class	15,095.191	.1042	4-class	8916.199	.2875
SHS/HSE 2012					
2-class	14,217.918	.0109			
3-class	13,759.639	**.0296**			
4-class	**13,692.114**	.0776			
SOGS			DSM-IV—Y/N		
BGPS 1999			APMS 2007		
2-class	10,819.723	<.0001	2-class	3412.344	<.0001
3-class	**10,728.964**	**.0031**	3-class	**3317.343**	**.0013**
4-class	10,805.677	.4121	4-class	3369.449	.1862

Statistics highlighted in bold identify which model is the best fit of the data

For one analysis (DSM-IV >0 scoring, BGPS 1999), indices also showed that a five class model was superior to a four class (LRT *p* < .05)

### Indices of Fit

Results from other indices of fit are reported below. An examination of AICs revealed that these tended to support a four class model for the DSM LCAs where individuals were dichotomised using the BGPS scoring scheme. Although similar to BIC and other indices, a four-class model was rejected as the third class was small (around thirty members) to begin with and the additional class further split this class. For the other LCAs (bar one exception) AIC supported six or more classes. These additional classes tended to split the second and third classes into smaller groups, leaving a class of a few hundred individuals (who overwhelmingly endorsed the preoccupation and loss-chasing items) and small classes with 30–40 members in each. In some cases (e.g. a number of the >0 DSM LCAs) this was readily interpretable. However in the majority of LCAs this was not the case; six-class solutions tended to produce a number of very small latent classes (<10 cases), and it appeared that these were largely spurious.

Classification accuracy was very high across the models, and was similar regardless of the number of classes specified. The entropy of the models changed very little between analyses, with a classification accuracy of approximately 0.9. Classification accuracy for the first class was slightly higher (around 0.95), and very similar for the second and third (around 0.9). This difference is not surprising given that the first class in each LCA contained several thousand individuals that did not endorse any indicator.

### Consistency Over Time

It should be noted there are important differences in sampling and elicitation between surveys. However, it is clear that bar one exception, LCAs of the same measure show notable consistency between survey years. Although we cannot definitively test this, and this should be taken with the caveat that these considerations are ultimately somewhat subjective, the estimated latent class models for assessments used on more than one occasion over the five surveys show notable similarity. Even in the latent classes with smaller sample sizes, these show the same pattern of responding. The one exception to this is the ordinal DSM-IV measure using the BGPS cutting score, which had a very small, inconsistent third class. For other DSM and PGSI LCAs (Figs. 3, 4), these indicate similar latent class models across the different surveys.

### Demographics and Gambling Behaviour

In the supplementary materials (Tables S21–S26) we report detailed descriptive statistics concerning demographic information and past-year prevalence on gambling between the latent classes. Comparisons between years are not considered because of market changes and different survey and item elicitation. Overall there are a number of cases (e.g. online gambling/betting, age of first gamble, scratchcard and slot machine play) where considerable differences between the first and second/third classes were observed, but not between the second and third classes. There were also a number of variables (e.g. smoking prevalence, wager amount/monthly spend, FOBT use) which graded alongside the severity of the classes. These paint a picture similar to the LCA indicators; some imply a continuum of severity, others show more marked differences between the second and third classes. There were some differences in gambling behaviour between assessments; in particular the PGSI and SOGS demonstrate higher prevalence of many gambling behaviours. This is likely because fewer individuals endorsed any of the indicators on these measures relative to the ordinal DSM measure. However, a consistent pattern between classes persists.

### Adapted DSM-IV Pathological Gambling Criteria (BGPS Series): BGPS Scoring

All four LCA’s indicated a three-class model (Table S2). However, fit indices only showed marginal differences between two and three-class models. The LRT’s supported a two-class model. Plotting the responses probabilities for two and three latent-class models revealed that two-class models (Fig. 1) were more consistent than three-class models (Fig. 2). The third class in three-class models varied considerably between samples, on some indicators differing by more than 80 %. However, in one instance there was evidence that local independence was violated; examination of the bivariate residuals suggested there was considerable residual covariance between indicators. Three-class models met this assumption. Consequently, although a three-class model was statistically a better fit of the data, the extra class did not show a consistent pattern of responding, likely due to the very low class size (*n* = 28, 29, 33, 10). Furthermore, none of the response probabilities for class three exceeded 0.75, suggesting these were weak indicators. This was worse for two-class models, where the highest endorsement probability (item 2) was 0.59. In addition it appeared, as discussed below, that the latent class model from this scoring method differed from other DSM based assessments analysed.

### Adapted DSM-IV Pathological Gambling Criteria (BGPS Series): Scores >0

BIC indices and LRTs supported a three-class solution (Table S3) in three analyses. For HSE 2012 data, indices supported a four-class solution, although LRTs supported a three-class model. This fourth class consisted of 10 cases in which respondents endorsed eight or more indicators. Comparisons with other cutoffs indicated this group comprised severe problem gamblers and gamblers likely to endorse many problem behaviours at low frequency.

Plots of the response probabilities (Fig. 3) and scores (Table S17) demonstrated a high level of consistency between samples. The recreational gambler subtype comprised almost all of the respondents who endorsed zero or one criteria, the intermediate group between two and four (or two and five in the BGPS 1999 analysis), and the third scores above 5 or 6. Recreational gamblers, where an indicator was likely to be endorsed, this was overwhelmingly the loss-chasing and preoccupation items. Endorsement rates for these criteria were similar for the intermediate and high severity groups. The intermediate groups had a high probability of endorsing the preoccupation and loss-chasing items, and a moderate to low probability of endorsing needing to gamble with more money to get the same feeling of excitement. Items measuring loss of control showed the largest differences between the two latent classes, with 80 % or more of the most severe gamblers endorsing these items, versus 15 % or so of intermediate gamblers. The final three items, probing consequences of pathological gambling, showed strong differences between the second and third classes, but endorsement probabilities were much lower; these showed fairly low endorsement by the highest severity group, and so while sufficient to discriminate between the two groups, this was not a necessary indicator of group membership in the manner the loss of control items appeared to be.

### Adapted DSM-IV Pathological Gambling Criteria (BGPS Series): Polytomous

BIC indices supported a three-class model. Three of four LRT’s supported a three-class model as well. The LRT of the HSE 2012 data supported a two-class model. Comparing the response probabilities for each latent class revealed that the latent class models were very similar to those with the >0 cutoff used. Examination of the group means (Figure S1) again revealed a very similar pattern to the response probabilities for the >0 cutoff (Fig. 3).

### Adapted DSM-IV Pathological Gambling Criteria (APMS 2007): Yes/No

The LCA supported a three-class model. The proportion the sample assigned to each latent class resembled the BGPS cutoff in class size. This revealed a group of recreational gamblers with minimal probability of endorsing any criterion. The second group showed low endorsement of multiple PG symptoms and higher probability of endorsing preoccupation and loss-chasing indicators. The third group had a high probability of endorsing every indicator with the exception of committing criminal acts to fund gambling. Comparing this LCA with other DSM measures (Figs. 2, 3) revealed that for the first seven criteria the data strongly resembled the three-class model found with the >0 cutoff, but for the remaining items, the pattern of symptom endorsement was more similar to the BGPS cutoffs. The intermediate class was consistent with both the >0 and BGPS cutoffs, as both demonstrated similar response patterns.

### PGSI Analyses

Two analyses of the PGSI data supported a three-class model and the third marginally supported a four-class model. All of the LRT’s supported a three-class model. The first class had minimal probability of endorsing any indicator. The second class had a high probability of endorsing two items (1 – betting more than one could afford to lose, and 3 – loss-chasing), and a moderate probability (between 0.2 and 0.4) of endorsing three of the indicators (2 – needing to gamble with more money to get same feeling of excitement, 7 – others criticizing gambling, and 9 – felt guilty about gambling). The third had a high probability (>0.7) for all items. However, overall severity remained moderate; item means were between 1.4 and 1.6, i.e. between ‘sometimes’ and ‘most of the time’. Between the second and third classes, high severity items identified by IRT analyses (items 4,6,8) (Miller et al. 2013), and three of the four items measuring loss of control (items 2,3,4,8) (Kincaid et al. 2013) showed considerable separation between classes (>0.8 for class 3, <0.2 for class 2). However, as these items overlap, it is difficult to adjudge between loss of control or severity explanations between latent classes. Item scores were consistent between classes (Fig. 4), and the distribution of PGSI scores (Table S17) were similar, indicating that the third class strongly resembled the PGSI category of problem gambler (8+).

### SOGS Analysis

The SOGS LCA supported a three-class model. The first class had a minimal probability of endorsing any of the indicators. The second class showed moderate (between 0.4 and 0.5) probability of endorsing two items: excessive betting and criticism about gambling not dissimilar to the second class in the PGSI LCA. This group had a lower (<30 %) probability of endorsing items querying borrowing household funds to gamble, feeling they might have a problem with gambling, loss-chasing and lying about winning. Comparing most likely class membership against SOGS scores closely resembled the interpretative categories of the SOGS (Table S18). However, there were very few strong indicators of latent class membership in the SOGS; item endorsement probability did not exceed 0.8 for any item across the three classes (Table S13), and the probability of endorsement exceeded 0.7 for only three: excessive gambling, guilt and other criticizing one’s gambling.

## Discussion

Analyses of disordered gambling from five nationally representative surveys revealed evidence for a three-class latent structure. The latent structure of these analyses was similar between assessments. The subtypes showed minimal overlap on assessment score, but indicators related to loss of control displayed the greatest differences between the medium and high severity latent classes. Furthermore, with one exception, analyses on the same assessment across time showed notable consistency. These findings are consistent with previous LCAs of DSM data, and extend to two frequent used assessments. Despite these assessments ostensibly measuring different conceptualizations of disordered gambling, they appear to converge on a common structure.

The analysis identified a combination of quantitative and qualitative differences between latent classes. The analyses indicated that the latent classes were ordered along a dimension of severity, as the scores of latent class members showed very little overlap between one another (Tables S15–S19). However, the greatest differences were observed on items relating to loss of control, a central construct in addiction, where there were typically high probabilities of endorsement (c. 80 %) for the highest severity class, and low probabilities of endorsement (c. 15 %) for the intermediate severity group (Fig. 3). This is potentially indicative of a difference in the type of symptoms different groups of gamblers endorsed rather than just the frequency, consistent with a qualitative distinction and is convergent with other latent structure analyses of disordered gambling data that identified categorical differences. This was the case with DSM and the SOGS items (where strong indicators were identified), but for PGSI loss of control and ‘difficult’ (i.e. high severity) items overlapped, meaning it wasn’t possible to discriminate between these competing explanations. It remains difficult to characterise disordered gamblers at the extreme end of a continuum, given the overall indicator distribution. Only in one instance did more than a quarter of individuals endorse at least one item. Even then, the indicators were very substantially skewed (James et al. 2014). If it can be plausibly claimed that problem gamblers form the extreme of a continuum, then a more sensitive measurement would be highly beneficial.

The third latent class of gamblers closely resembled the taxon previously observed in taxometric analyses of disordered gambling assessments. Taxometric studies identified a qualitatively distinct category of very high severity gamblers on DSM and PGSI measurements (James et al. 2014; Kincaid et al. 2013). The present results converge with these findings. It should be noted that response probabilities for these items revealed that the largest differences were on items related to loss of control, not the highest severity items. In some cases it does appear that the boundary where this third class emerges is very slightly lower severity than the one identified by taxometric analysis. The LCA’s found that the highest severity category used in the PGSI (8+) was closely calibrated to the lowest score at which cases were assigned to the third latent class. None of the analyses indicated that the original (1–2/3–7) or modified (1–3/4–7) intermediate sub-categories formed distinct latent classes. Previous studies failed to find differences for the original categories (Currie et al. 2013). However, this might be due to the low number of non-zero responses on the PGSI. It might be useful to combine these data to test whether the presence of intermediate categories might be detected with a larger dataset.

Previous taxonomies of disordered gambling have identified the presence of three categories of gamblers across the general population: Shaffer, Hall, and Vander Bilt (1999) for instance outline a standardisation of terminology for, identifying three levels of disordered gambling. Level one gamblers consist of recreational or non-gamblers, level two gamblers display subclinical difficulties with gambling, and level three gamblers meet clinical criteria for Gambling Disorder or Pathological Gambling. The findings of these analyses appear to strongly support such a demarcation, both in the number of groupings identified and the types of behaviours members of the identified latent classes are likely to endorse.

These results inform a wider debate concerning the reclassification of Gambling Disorder in the DSM-5. The manual makes three major alterations from the conceptualisation of Pathological Gambling in the DSM-IV; one criterion was removed (engaging in criminal acts to fund gambling), the clinical cutoff was reduced from five criteria to four, and it implemented a more graded approach to classifying disordered gamblers, distinguishing between low, moderate and high severity. These findings suggest that moderate and severely disordered gamblers form a distinct latent class from other disordered and (non-clinical) problem gamblers. In addition, the results demonstrate that the removal of the illegal acts criterion ought to make very little difference of the ability of the criteria to distinguish between different levels of gambling problems, in line with the rationale for removing this criterion. However, concerns have been raised that although removing this item is beneficial for prevalence research as the item shows minimal incremental validity, this might shape clinical practices in a manner that might be counterproductive (Bowden-Jones 2013). There are two other criteria that behave in a similar manner across studies, but more importantly between the moderate and high severity gamblers there are other items that discriminate these groups more comprehensively.

Analysis of the SOGS data indicated that this assessment measures a similar latent structure to the other screens in this report. It appears that gamblers in the second/intermediate latent class endorse relatively similar items across measurements as well. The scores for latent class members closely resembled the three subtypes for the SOGS. Although of declining importance in population assessment (Williams et al. 2012), this finding remains of interest as the SOGS is widely used in experimental research.

The cutoff’s used in the BGPS DSM measure did not produce consistent results for the highest severity latent class. Endorsement probabilities of PG behaviours varied between samples in contrast to the other measures. BIC indices for two and three-class models were consistently close to one another; LRT’s conducted on the latent class model supported a two-class model. This cutoff was used in an analysis that found that UK PG prevalence increased between 2007 and 2010. The report itself (Wardle et al. 2011) and the present analysis highlight that this should be taken with caution. Although comparisons between gambling and health surveys should be made with caution as survey framing affects responding (Williams et al. 2012), the DSM cutoff used in the BGPS/HSE surveys produced similar levels of endorsement to the APMS measure but did not demonstrate similar levels of disordered gambling prevalence (Table 1). It might be of benefit to pool these data to compare class membership between samples in a similar manner to the BGPS analysis (Wardle et al. 2011).

An important caveat is that while these findings identify a common latent structure in measurements of problem gambling, it is not possible to claim this generalises to other jurisdictions. As the analysis was restricted to British gamblers, these results may not translate to other countries where different restrictions on gambling or other circumstances prevail. However, there is some cause for optimism in this regard. Studies in the USA and South Africa have found commensurate results under different conditions; in the USA, LCA of NESARC data based off a structured interview revealed a similar pattern of results, and taxometric analysis of South African data identified a distinct latent class (albeit with much higher prevalence than UK/USA) in PGSI data.

To conclude, seventeen LCAs of disordered gambling assessment data revealed a consistent three-class structure in which gamblers differed in severity, and that clusters of disordered gambling indicators (loss-chasing/preoccupation, loss of control) characterised class membership. This final group appeared to show qualitative differences from the other latent classes on the basis that items measuring a loss of control showed the greatest differences between the latent classes. Our analyses of these large-scale surveys suggest that research on the transition from recreational to disordered gambling should focus on the factors that make individuals susceptible to loss of control. These may be internal to the individual, such as impulsivity; external to the individual, such as the schedules of reinforcement of the gambling games, or an interaction between the two.

## Electronic supplementary material

Below is the link to the electronic supplementary material.

Supplementary material 1 (TIFF 1150 kb)

Supplementary material 2 (DOCX 107 kb)

## Footnotes

1 Item 16 has 11 sub-items. Items 1, 2, 3, 12, 16j and 16k aren’t counted towards the SOGS score.

2 Please see supplementary information for details on sampling.

3 The BGPS 1999 data was weighted and adjusted for clustering; no stratification variable was included in the dataset.
