# Supplementary figures and images for: Loss of Control as a Discriminating Factor Between Different Latent Classes of Disordered Gambling Severity
Source: J Gambl Stud. 2016 Feb 18;32(4):1155–73. doi: 10.1007/s10899-016-9592-z (PMC5101294; doi:10.1007/s10899-016-9592-z)

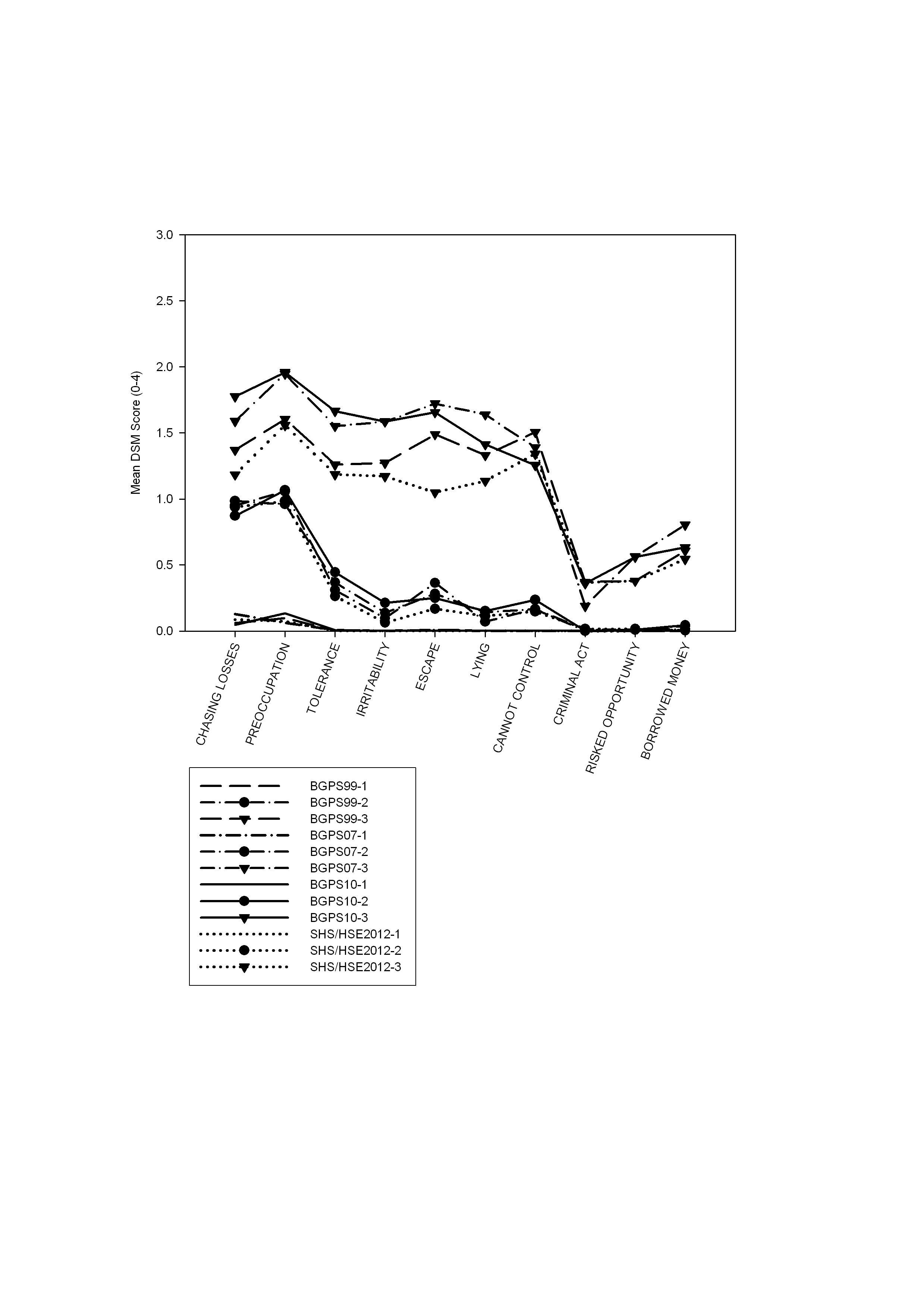

Supplement: Supplementary file 1 — Supplementary material 1 (TIFF 1150 kb) [file 10899_2016_9592_MOESM1_ESM.tif]
