# Supplementary material for: Loss of Control as a Discriminating Factor Between Different Latent Classes of Disordered Gambling Severity
Source: J Gambl Stud. 2016 Feb 18;32(4):1155–73. doi: 10.1007/s10899-016-9592-z (PMC5101294; doi:10.1007/s10899-016-9592-z)
Supplement: Supplementary file 2 — Supplementary material 2 (DOCX 107 kb) [file 10899_2016_9592_MOESM2_ESM.docx]

Table S1. Problem gambling assessments administered to respondents in the surveys included in the present analysis.

| *Problem Gambling Severity Index (BGPS 2007, BGPS 2010, SHS & HSE 2012)*  Responses (all items) – Never (0), sometimes (1), most of the time (2), almost always (3). |
| --- |
| 1. Have you bet more than you could really afford to lose? |
| 2. Have you needed to gamble with larger amount of money to get the same feeling of excitement? |
| 3. When you gambled, did you go back another day to try to win back the money you lost? * - Presented in the BGPS series as: Have you gone back to try to win back the money you’d lost? |
| 4. Have you borrowed money or sold anything to get money to gamble? |
| 5. Have you felt you might have a problem with gambling? |
| 6. Has gambling caused you any health problems, including stress or anxiety?  * Presented in the BGPS series as: Have you felt that gambling has caused you any health problems, including stress or anxiety? |
| 7. Have people criticized your betting or told you that you had a gambling problem, regardless of whether or not you thought it was true? |
| 8. Has your gambling caused any financial problems for you or your household?  * Presented in the BGPS series as: Have you felt your gambling has caused financial problems for you or your household? |
| 9. Have you felt guilty about the way you gamble or what happens when you gamble? |
|  |
|  |

Table S1 (continued)

| *DSM-IV Pathological Gambling Criteria (BGPS series and HSE 2012)*  Responses: Item 1: Never (0), Some of the time (less than half the time I lost) (1), Most of the time I lost (2), Every time I lost (3)  Items 2 – 10: Never (0), Occasionally (1), Fairly Often (2), Very Often (3) |
| --- |
| 1. When you gamble, how often do you go back another day to win back money you lost? |
| 2. How often have you found yourself thinking about gambling (that is relieving past gambling experiences, planning the next time you will play, or thinking of ways to get money to gamble)? |
| 3. Have you needed to gamble with more and more money to get the excitement you are looking for? |
| 4. Have you felt restless or irritable when trying to cut down gambling? |
| 5. Have you gambled to escape from problems or when you are feeling depressed, anxious or bad about yourself? |
| 6. Have you lied to family, or others, to hide the extent of your gambling? |
| 7. Have you made unsuccessful attempts to control, cut back or stop gambling? |
| 8. Have you committed a crime in order to finance gambling or pay gambling debts? |
| 9. Have you risked or lost an important relationship, job, educational or work opportunity because of gambling? |
| 10. Have you asked others to provide money to help with a desperate financial situation caused by gambling? |
|  |
| *DSM-IV Pathological Gambling Criteria (APMS 2007)*  Responses (all items): Yes/No |
| 1. Are you preoccupied with gambling (e.g. preoccupied with reliving past gambling experiences or planning the next venture, or thinking of ways to get money with which to gamble)? |
| 2. Do you need to gamble with increasing amounts of money in order to achieve the desired excitement? |
| 3. Have you made repeated unsuccessful attempts to control, cut back or stop gambling? |
| 4. Are you restless when attempting to cut down or stop gambling?  * Q4 only asked if answer to Q3 was ‘YES’. |
| 5. Do you gamble as a way of escaping from problems or relieving feelings of helplessness, guilt, anxiety or depression? |
| 6. After losing money gambling, do you often return another day to get even? |
| 7. Do you lie to family members, therapists, or to others to conceal the extent of involvement with gambling? |
| 8. Have you committed illegal acts such as forgery, fraud, theft or embezzlement to finance gambling? |
| 9. Have you jeopardised or lost a significant relationship, job, or educational or career opportunity because of gambling? |
| 10. Do you rely on others to provide money to relieve a desperate financial situation caused by gambling? |

Table S1 (continued)

| *South Oaks Gambling Screen (BGPS 1999)* – Unless stated, responses are yes/no. This only includes items that contribute to the calculated SOGS score. When responses that contribute to the SOGS score are not ‘YES’, they are highlighted in bold. Please note than several items are slightly different, either for purpose of brevity or adapted for a British population. |
| --- |
| 4. When you gamble, how often do you go back another day to win back money you have lost?  Responses: “Never”, “Some of the time (less than half the time I lose)”, “**Most of the times I lose”, “Every time I lose”** |
| 5. Have you ever claimed to be winning money gambling, but weren’t really? In fact, you lost?  Presented as: Have you claimed to be winning money from gambling when in fact, you lost?  Responses: “Never”, **“Yes, less than half the time I lost”, “Yes, most of the time”** |
| 7. Did you ever gamble more than you intended to?  Presented as: Do you spend more time or money gambling than you intended? |
| 8. Have people criticized your betting or told you that you had a problem, regardless of whether or not you thought it was true?  Presented as: Have people criticized your gambling? |
| 9. Have you ever felt guilty about the way you gamble, or what happens when you gamble? |
| 10. Have you ever felt like you would like to stop betting money on gambling, but didn’t think you could? |
| 11. Have you ever hidden betting slips, lottery tickets, gambling money, IOU’s, or other signs of betting or gambling from your spouse, children or other important people in your life? |
| 13. (If answered “Yes” to 12: Have you ever argued with people you live with over how you handle money?) Have these arguments ever centred on your gambling? |
| 14. Have you ever borrowed from someone and not paid them back as a result of your gambling? |
| 15. Have you ever lost time from work (or school) due to betting money or gambling? |
| 16. If you borrowed money to gamble or pay gambling debts, who or where did you borrow money from: |
| 16a. From household money |
| 16b. From your spouse |
| 16c. From other relatives or in-laws |
| 16d. From banks, loan companies or credit unions  Presented as: banks, building societies, loan companies, or credit companies |
| 16e. From credit cards |
| 16f. From loan sharks |
| 16g. You cashed in stocks, bonds or other securities |
| 16h. You sold personal or family property. |
| 16i. You borrowed on your checking accounts (passed bad cheques). |
| 6. Do you feel you have ever had a problem with betting or money gambling?  Responses: “No”, **“Yes”, “Yes, in the past, but not now”**  ***** Presented as Yes/No in the BGPS 1999, as adapted for past year prevalence. |

Table S2. Indices of model fit for weighted LCA’s conducted on the modified version of the DSM-IV Pathological Gambling Criteria used in the BGPS, scored using the cutoffs used in the BGPS reports (symptoms classified as present – items 1 – 7 > 1, items 8 – 10 – > 0).

| Classes | Log-likelihood | AIC | BIC | SSABIC | Entropy | LMR-LRT *p* | VLMR-LRT *p* |
| --- | --- | --- | --- | --- | --- | --- | --- |
| *BGPS 1999* | |  |  |  |  |  |  |
| 1-class | -2472.021 | 4964.042 | 5029.715 | 4997.939 | - | - | - |
| 2-class | -1849.968 | 3741.935 | 3879.849 | 3813.118 | 0.99 | <.0001 | <.0001 |
| 3-class | -1798.731 | 3661.463 | **3871.617** | **3769.931** | 0.967 | .0104 | .0099 |
| 4-class | -1773.361 | **3632.722** | 3915.116 | 3778.476 | 0.97 | **.0327** | **.0343** |
| 5-class | -1762.833 | 3633.667 | 3988.302 | 3816.708 | 0.972 | .4925 | .4879 |
| 6-class | -1753.605 | 3637.21 | 4064.085 | 3857.536 | 0.973 | .5103 | .5072 |
|  |  |  |  |  |  |  |  |
| *BGPS 2007* | |  |  |  |  |  |  |
| 1-class | -3272.314 | 6564.627 | 6630.758 | 6598.981 | - | - | - |
| 2-class | -2557.443 | 5156.886 | 5295.76 | 5229.029 | **0.994** | **<.0001** | **<.0001** |
| 3-class | -2508.816 | 5081.632 | **5293.249** | **5191.563** | 0.974 | 0.1013 | 0.0989 |
| 4-class | -2490.336 | **5066.671** | 5351.032 | 5214.392 | 0.984 | 0.2883 | 0.2846 |
| 5-class | -2484.135 | 5076.271 | 5433.375 | 5261.78 | 0.993 | 0.5255 | 0.5246 |
| 6-class | -2472.895 | **5075.79** | 5505.638 | 5299.088 | 0.852 | 0.4904 | 0.4899 |
|  |  |  |  |  |  |  |  |
| *BGPS 2010* | |  |  |  |  |  |  |
| 1-class | -3748.56 | 7517.121 | 7583.601 | 7551.824 | - | - | - |
| 2-class | -2831.913 | 5705.827 | 5845.435 | 5778.704 | 0.984 | **<.0001** | **<.0001** |
| 3-class | -2771.551 | 5607.102 | **5819.839** | **5718.153** | 0.958 | 0.1708 | 0.1676 |
| 4-class | -2745.868 | 5577.736 | 5863.602 | 5726.961 | 0.972 | 0.5028 | 0.5007 |
| 5-class | -2727.647 | 5563.294 | 5922.289 | 5750.693 | 0.969 | 0.1712 | 0.1703 |
| 6-class | -2715.692 | **5561.385** | 5993.508 | 5786.957 | 0.912 | 0.4336 | 0.4325 |
|  |  |  |  |  |  |  |  |
| *HSE 2012 & SHS 2012* | |  |  |  |  |  |  |
| 1-class | -2885.033 | 5790.066 | 5858.472 | 5826.694 | - | - | - |
| 2-class | -2233.364 | 4508.728 | 4652.381 | 4585.648 | 0.992 | **<.0001** | **<.0001** |
| 3-class | -2179.977 | 4423.954 | **4642.852** | **4541.165** | 0.984 | 0.2627 | 0.2581 |
| 4-class | -2159.378 | **4404.755** | 4698.9 | 4562.256 | 0.988 | 0.502 | 0.4999 |
| 5-class | -2143.427 | 4394.855 | 4764.246 | 4592.647 | 0.982 | 0.5484 | 0.5474 |
| 6-class | -2122.869 | 4375.738 | 4820.376 | 4613.821 | 0.983 | 0.3493 | 0.3486 |

Table S3. Indices of model fit for weighted LCA’s conducted on the modified version of the DSM-IV Pathological Gambling Criteria used in the BGPS, classifying respondents who gave a response other than ‘Never’ (0) to any of the items as displaying a symptom present.

| Classes | Log-likelihood | AIC | BIC | SSABIC | Entropy | LMR-LRT *p* | VLMR-LRT *p* |
| --- | --- | --- | --- | --- | --- | --- | --- |
| *BGPS 1999* | |  |  |  |  |  |  |
| 1-class | -7401.198 | 14102.395 | 14168.068 | 14136.291 | - | - | - |
| 2-class | -5706.346 | 11454.692 | 11592.606 | 11525.875 | **0.963** | <.0001 | <.0001 |
| 3-class | -5527.124 | 11118.248 | **11328.402** | 11226.717 | 0.93 | **<.0001** | **<.0001** |
| 4-class | -5487.991 | 11061.982 | 11344.377 | **11207.737** | 0.915 | 0.1449 | 0.1421 |
| 5-class | -5473.252 | 11054.503 | 11409.138 | 11237.544 | 0.925 | **0.0313** | **0.0303** |
| 6-class | **-5458.301** | **11046.602** | 11473.748 | 11266.929 | 0.87 | 0.0948 | 0.0929 |
|  |  |  |  |  |  |  |  |
| *BGPS 2007* | |  |  |  |  |  |  |
| 1-class | -8013.933 | 16047.865 | 16113.996 | 16082.219 | - | - | - |
| 2-class | -6553.48 | 13148.96 | 13287.834 | 13221.102 | **0.944** | <.0001 | <.0001 |
| 3-class | -6339.104 | 12742.208 | **12953.825** | 12852.139 | 0.891 | **.0004** | **.0004** |
| 4-class | -6300.403 | 12686.807 | 12971.168 | **12834.527** | 0.864 | .087 | .085 |
| 5-class | -6271.074 | 12650.149 | 13007.253 | 12835.658 | 0.901 | .3794 | .3766 |
| 6-class | -6250.972 | **12631.944** | 13061.792 | 12855.242 | 0.923 | .3546 | .3526 |
|  |  |  |  |  |  |  |  |
| *BGPS 2010* | |  |  |  |  |  |  |
| 1-class | -9670.177 | 19360.354 | 19426.835 | 19395.058 | - | - | - |
| 2-class | -7689.533 | 15421.067 | 15560.675 | 15493.944 | 0.929 | <.0001 | <.0001 |
| 3-class | -7404.648 | 14873.296 | **15086.033** | 14984.347 | 0.888 | **.0007** | **.0007** |
| 4-class | -7361.663 | 14809.325 | 15095.191 | **14958.55** | 0.889 | .1042 | .1021 |
| 5-class | -7342.02 | **14792.039** | 15151.034 | 14979.438 | 0.904 | .5528 | .5509 |
| 6-class | -7332.073 | 14794.146 | 15226.269 | 15019.718 | 0.9 | .6828 | .6824 |
|  |  |  |  |  |  |  |  |
| *HSE 2012 & SHS 2012* | | |  |  |  |  |  |
| 1-class | -8702.537 | 17425.074 | 17493.48 | 17461.703 | - | - | - |
| 2-class | -7016.133 | 14074.266 | 14217.918 | 14151.185 | 0.952 | .0109 | .0101 |
| 3-class | -6738.368 | 13540.736 | 13759.639 | 13657.946 | 0.905 | **.0296** | **.0288** |
| 4-class | -6655.985 | 13397.969 | **13692.114** | **13555.47** | 0.902 | .0776 | .0764 |
| 5-class | -6630.339 | 13368.678 | 13738.07 | 13566.47 | 0.918 | .2578 | .2557 |
| 6-class | -6610.379 | **13350.758** | 13795.396 | 13588.841 | 0.933 | 0.4225 | 0.4214 |

Table S4. Indices of model fit for weighted LCA’s conducted on the modified version of the DSM-IV Pathological Gambling Criteria used in the BGPS series, in the polytomous format the assessments was administered in.

| Classes | Log-likelihood | AIC | BIC | SSABIC | Entropy | LMR-LRT *p* | VLMR-LRT *p* |
| --- | --- | --- | --- | --- | --- | --- | --- |
| *BGPS 1999* | |  |  |  |  |  |  |
| 1-class | -8264.801 | 16589.601 | 16786.621 | 16691.291 | - | - | - |
| 2-class | -6857.508 | 13837.015 | 14237.622 | 14043.784 | **0.969** | <.0001 | <.0001 |
| 3-class | -6634.07 | 13452.141 | **14056.334** | **13763.988** | 0.927 | **.0038** | **.0037** |
| 4-class | -6575.468 | 13396.935 | 14204.715 | 13813.861 | 0.926 | .7766 | .7766 |
| 5-class | -6530.489 | 13368.978 | 14380.345 | 13890.983 | 0.937 | .7856 | .7856 |
| 6-class | -6500.12 | **13370.239** | 14585.192 | 13997.323 | 0.937 | .7603 | .7603 |
|  |  |  |  |  |  |  |  |
| *BGPS 2007* | |  |  |  |  |  |  |
| 1-class | -9643.828 | 19347.655 | 19546.047 | 19450.716 | - | - | - |
| 2-class | -8099.425 | 16320.849 | 16724.245 | 16530.406 | **0.957** | <.0001 | <.0001 |
| 3-class | -7785.486 | 15754.972 | **16363.372** | 16071.025 | 0.915 | **.0278** | **.0272** |
| 4-class | -7687.598 | 15621.196 | 16434.601 | **16043.745** | 0.901 | .762 | .762 |
| 5-class | -7642.304 | 15592.609 | 16611.018 | 16121.654 | 0.914 | .8478 | .8478 |
| 6-class | -7609.158 | **15588.317** | 16811.731 | 16223.858 | 0.899 | .7619 | .7619 |
|  |  |  |  |  |  |  |  |
| *BGPS 2010* | |  |  |  |  |  |  |
| 1-class | -11590.228 | 23240.456 | 23439.897 | 23344.566 | - | - | - |
| 2-class | -9536.282 | 19194.564 | 19600.095 | 19406.255 | 0.946 | <.0001 | <.0001 |
| 3-class | -9164.643 | 18513.285 | **19124.905** | 18832.557 | 0.914 | **.0026** | **.0025** |
| 4-class | -9059.587 | 18365.174 | 19182.884 | **18792.026** | 0.894 | .7866 | .7866 |
| 5-class | -9019.424 | 18346.849 | 19370.648 | 18881.281 | 0.905 | .7889 | .7889 |
| 6-class | -8986.905 | **18343.81** | 19573.699 | 18985.824 | 0.898 | .7626 | .7626 |
|  |  |  |  |  |  |  |  |
| *HSE 2012 & SHS 2012* | | |  |  |  |  |  |
| 1-class | -10099.83 | 20259.66 | 20464.877 | 20369.544 | - | - | - |
| 2-class | -8353.352 | 16828.703 | 17245.979 | 17052.135 | 0.965 | **<.001** | **<.001** |
| 3-class | -8033.812 | 16251.624 | **16880.958** | 16588.603 | 0.912 | 0.7259 | 0.7255 |
| 4-class | -7915.787 | 16077.575 | 16918.966 | **16528.101** | 0.917 | 0.7699 | 0.7698 |
| 5-class | -7838.602 | 15985.204 | 17038.653 | 16549.278 | 0.927 | 0.7608 | 0.7608 |
| 6-class | -7788.164 | **15946.327** | 17211.835 | 16623.948 | 0.939 | 0.769 | 0.769 |

Table S5. Indices of model fit for weighted polytomous LCA’s conducted on Problem Gambling Severity Index data from the BGPS 2007, 2010 and SHS & HSE 2012.

| Classes | Log-likelihood | AIC | BIC | SSABIC | Entropy | LMR-LRT *p* | VLMR-LRT *p* |
| --- | --- | --- | --- | --- | --- | --- | --- |
| *BGPS 2007* | |  |  |  |  |  |  |
| 1-class | -6446.533 | 12947.067 | 13125.98 | 13040.182 | - | - | - |
| 2-class | -4751.487 | 9612.975 | 9977.427 | 9802.654 | **0.981** | **.0032** | **.0031** |
| 3-class | -4481.076 | 9128.152 | **9678.144** | 9414.395 | 0.967 | .1543 | .1551 |
| 4-class | -4362.823 | 8947.646 | 9683.177 | **9330.453** | 0.957 | .828 | .828 |
| 5-class | -4324.576 | 8927.152 | 9848.222 | 9406.523 | 0.965 | .7723 | .7723 |
| 6-class | -4293.064 | **8920.128** | 10026.063 | 9496.063 | 0.968 | .7606 | .7606 |
|  |  |  |  |  |  |  |  |
| *BGPS 2010* | |  |  |  |  |  |  |
| 1-class | -7425.183 | 14904.366 | 15083.892 | 14998.094 | - | - | - |
| 2-class | -5431.798 | 10973.596 | 11339.296 | 11164.523 | 0.976 | <.0001 | <.0001 |
| 3-class | -5135.229 | 10436.458 | 10988.334 | 10724.584 | 0.95 | **.0222** | **.0217** |
| 4-class | -5013.377 | 10248.755 | **10986.805** | **10634.079** | 0.945 | .7769 | .7768 |
| 5-class | -4955.385 | 10188.771 | 11112.996 | 10671.295 | 0.964 | .7603 | .7603 |
| 6-class | -4908.076 | **10150.153** | 11260.552 | 10729.876 | 0.941 | .7659 | .7659 |
|  |  |  |  |  |  |  |  |
| *HSE 2012 & SHS 2012* | | |  |  |  |  |  |
| 1-class | -5938.988 | 11931.977 | 12116.836 | 12031.037 | - | - | - |
| 2-class | -4255.934 | 8621.869 | 8998.434 | 8823.657 | 0.986 | **<.0001** | **<.0001** |
| 3-class | -4051.545 | 8269.091 | **8837.362** | 8573.607 | 0.984 | .0662 | .0652 |
| 4-class | -3967.105 | 8156.211 | 8916.199 | **8563.456** | 0.986 | .2875 | .2865 |
| 5-class | -3932.543 | 8143.086 | 9094.769 | 8653.059 | 0.97 | .8062 | .8062 |
| 6-class | -3896.419 | **8126.839** | 9270.228 | 8739.541 | 0.971 | .7611 | .7611 |

Table S6. Indices of model fit for weighted LCA’s conducted on the South Oaks Gambling Screen from BGPS 1999 data, and an assessment derived from the DSM-IV Pathological Gambling criteria, administered in a Yes/No format in the APMS 2007.

| Classes | Log-likelihood | AIC | | BIC | | SSABIC | | Entropy | | LMR-LRT *p* | | VLMR-LRT *p* | |  |
| --- | --- | --- | --- | --- | --- | --- | --- | --- | --- | --- | --- | --- | --- | --- |
| *BGPS 1999 - SOGS* | | | |  | |  | |  | |  | |  |  |  |
| 1-class | -6564.075 | | 13168.15 | | 13298.53 | | 13234.977 | | - | | - | | - | |
| 2-class | -5235.222 | | 10552.445 | | 10819.723 | | 10689.44 | | **0.966** | | <.0001 | | <.0001 | |
| 3-class | -5100.393 | | 10324.786 | | **10728.964** | | **10531.949** | | 0.94 | | **.0031** | | **.0028** | |
| 4-class | -5049.3 | | 10264.601 | | 10805.677 | | 10541.932 | | 0.942 | | .4121 | | .4095 | |
| 5-class | -5011.744 | | 10231.487 | | 10909.462 | | 10578.986 | | 0.958 | | .1983 | | .1964 | |
| 6-class | -4984.704 | | **10219.409** | | 11034.283 | | 10637.076 | | 0.956 | | .5296 | | .5278 | |
|  |  | |  | |  | |  | |  | |  | |  | |
| *APMS 2007 – DSM (Y/N)* | | | |  | |  | |  | |  |  |  |  |  |
| 1-class | -2214.985 | | 4449.969 | | 4511.767 | | 4479.992 | | - | | - | | - | |
| 2-class | -1620.285 | | 3282.569 | | 3412.344 | | 3345.617 | | **0.983** | | <.0001 | | <.0001 | |
| 3-class | -1527.795 | | 3119.591 | | **3317.343** | | **3215.663** | | 0.959 | | **.0013** | | **.0012** | |
| 4-class | -1508.86 | | 3103.719 | | 3369.449 | | 3232.817 | | 0.962 | | .1862 | | .1812 | |
| 5-class | -1489.371 | | 3086.742 | | 3420.449 | | 3248.865 | | 0.981 | | .3126 | | .2934 | |
| 6-class | -1476.972 | | **3083.091** | | 3485.627 | | 3279.091 | | 0.986 | | .2013 | | .1996 | |

Table S7. Response probabilities for two-latent class models based on an assessment derived from the DSM-IV Pathological Gambling Criteria used in the BGPS series, scored using the cutoff’s from the BGPS series (scores 0-3, 1-7 > 1, 8-10 > 0). Classes are ordered by severity/size of group membership, and standard errors are reported in brackets.

|  | BGPS 1999 | | BGPS 2007 | | | BGPS 2010 | | | HSE 2012 | | |  |
| --- | --- | --- | --- | --- | --- | --- | --- | --- | --- | --- | --- | --- |
|  | L1 (98.65%) | L2 (1.35%) | | L1 (98.75%) | L2 (1.25%) | | L1 (98.05%) | L2 (1.95%) | | L1 (98.9%) | L2 (1.1%) | |
| 1 | 0.019 (.002) | 0.416 (.071) | | 0.052 (.004) | 0.49 (.073) | | 0.019 (.002) | 0.518 (.063) | | 0.024 (.003) | 0.387 (.082) | |
| 2 | 0.012 (.002) | 0.601 (.072) | | 0.011 (.002) | 0.635 (.07) | | 0.021 (.003) | 0.633 (.061) | | 0.007 (.001) | 0.499 (.087) | |
| 3 | 0.001 (.001) | 0.355 (.073) | | 0.001 (0) | 0.444 (.07) | | 0.003 (.001) | 0.458 (.066) | | 0.001 (.001) | 0.363 (.09) | |
| 4 | 0 (0) | 0.315 (.073) | | 0 (0) | 0.468 (.078) | | 0.001 (.001) | 0.457 (.081) | | 0 (0) | 0.252 (.067) | |
| 5 | 0.001 (.001) | 0.504 (.089) | | 0.002 (.001) | 0.522 (.091) | | 0.001 (0) | 0.364 (.075) | | 0.001 (0) | 0.296 (.073) | |
| 6 | 0 (0) | 0.321 (.072) | | 0 (0) | 0.465 (.081) | | 0.000 (0) | 0.267 (.066) | | 0.001 (.001) | 0.448 (.096) | |
| 7 | 0.001 (.001) | 0.396 (.086) | | 0.002 (.001) | 0.32 (.071) | | 0.003 (.001) | 0.281 (.057) | | 0.002 (.001) | 0.355 (.081) | |
| 8 | 0 (0) | 0.185 (.056) | | 0 (0) | 0.101 (.043) | | 0 (0) | 0.147 (.044) | | 0 (0) | 0.261 (.08) | |
| 9 | 0 (0) | 0.239 (.06) | | 0 (0) | 0.28 (.073) | | 0.001 (0) | 0.244 (.057) | | 0.001 (0) | 0.304 (.086) | |
| 10 | 0.001 (0) | 0.346 (.078) | | 0.002 (.001) | 0.465 (.081) | | 0.002 (.001) | 0.354 (.067) | | 0.001 (0) | 0.393 (.094) | |

Table S8. Response probabilities for three latent class model for the adapted version of the DSM-IV Pathological Gambling Criteria used in the BGPS series, scored using the cutoff’s recommended in the BGPS series (scores 0-3, 1-7 > 1, 8-10 > 0). Latent classes are ordered by severity/group membership, and standard errors are reported in brackets.

|  |  | 1 | 2 | 3 | 4 | 5 | 6 | 7 | 8 | 9 | 10 |
| --- | --- | --- | --- | --- | --- | --- | --- | --- | --- | --- | --- |
| BGPS 1999 | 1 (97.59%) | 0.017 (.002) | 0.007 (.003) | 0.001 (.001) | 0 (0) | 0.001 (.001) | 0 (0) | 0.001 (.001) | 0 (0) | 0 (0) | 0.001 (0) |
|  | 2 (1.85%) | 0.3 (.087) | 0.585 (.107) | 0.106 (.054) | 0.107 (.052) | 0.184 (.067) | 0.05 (.037) | 0.092 (.046) | 0 (0) | 0.014 (.015) | 0.049 (.031) |
|  | 3 (0.56%) | 0.451 (.109) | 0.452 (.109) | 0.565 (.111) | 0.446 (.1) | 0.727 (.096) | 0.626 (.1) | 0.698 (.104) | 0.441 (.121) | 0.53 (.115) | 0.692 (.108) |
| BGPS 2007 | 1 (97.88%) | 0.049 (.004) | 0.007 (.002) | 0 (0) | 0.001 (0) | 0.001 (.001) | 0 (0) | 0.002 (.001) | 0 (0) | 0 (0) | 0.002 (.001) |
|  | 2 (1.58%) | 0.426 (.071) | 0.55 (.094) | 0.177 (.056) | 0.126 (.047) | 0.08 (.068) | 0.106 (.066) | 0.045 (.028) | 0.028 (.02) | 0.101 (.049) | 0.179 (.064) |
|  | 3 (0.53%) | 0.587 (.112) | 0.637 (.097) | 0.61 (.125) | 0.761 (.136) | 1 (0) | 0.788 (.082) | 0.648 (.128) | 0.154 (.081) | 0.407 (.116) | 0.625 (.131) |
| BGPS 2010 | 1 (96.57%) | 0.015 (.002) | 0.016 (.002) | 0.002 (.001) | 0.001 (.001) | 0 (0) | 0 (0) | 0.003 (.001) | 0 (0) | 0 (0) | 0.002 (.001) |
|  | 2 (2.83%) | 0.373 (.082) | 0.466 (.093) | 0.214 (.07) | 0.188 (.068) | 0.137 (.07) | 0.07 (.042) | 0.102 (.044) | 0.036 (.023) | 0.091 (.05) | 0.145 (.057) |
|  | 3 (0.6%) | 0.684 (.126) | 0.808 (.116) | 0.81 (.111) | 0.847 (.118) | 0.712 (.102) | 0.642 (.153) | 0.594 (.146) | 0.362 (.137) | 0.482 (.142) | 0.657 (.132) |
| HSE & SHS  2012 | 1 (98.51%) | 0.023 (.003) | 0.005 (.001) | 0 (.001) | 0 (0) | 0.001 (0) | 0.001 (.001) | 0.002 (.001) | 0 (0) | 0 (0) | 0 (0) |
|  | 2 (1.25%) | 0.335 (.086) | 0.423 (.097) | 0.172 (.062) | 0.166 (.062) | 0.208 (.066) | 0.279 (.086) | 0.273 (.084) | 0.048 (.032) | 0.08 (.053) | 0.166 (.066) |
|  | 3 (0.23%) | 0.479 (.166) | 0.578 (.186) | 0.837 (.088) | 0.355 (.15) | 0.379 (.155) | 0.727 (.176) | 0.401 (.164) | 0.961 (.031) | 1 (0) | 1 (0) |

Table S9. Response probabilities for the adapted version of the DSM-IV Pathological Gambling Criteria, with cutoff’s of any behaviour endorsed more than ‘never’. Three (and four in the case of the SHS & HSE 2012 data) class solutions. Latent classes are ordered by severity/group membership, and standard errors are reported in brackets.

|  | Class | 1 | 2 | 3 | 4 | 5 | 6 | 7 | 8 | 9 | 10 |
| --- | --- | --- | --- | --- | --- | --- | --- | --- | --- | --- | --- |
| BGPS 1999 | 1 (92.31%) | 0.046 (.004) | 0.09 (.006) | 0.004 (.001) | 0 (0) | 0.013 (.002) | 0 (0) | 0.001 (.001) | 0 (0) | 0 (0) | 0 (0) |
|  | 2 (6.69%) | 0.625 (.043) | 0.796 (.036) | 0.265 (.035) | 0.096 (.022) | 0.274 (.031) | 0.067 (.02) | 0.128 (.025) | 0 (0) | 0.009 (.005) | 0.013 (.007) |
|  | 3 (1.00%) | 0.855 (.055) | 0.892 (.049) | 0.778 (.066) | 0.789 (.071) | 0.867 (.064) | 0.914 (.053) | 0.912 (.054) | 0.246 (.076) | 0.296 (.076) | 0.467 (.102) |
| BGPS 2007 | 1 (88.95%) | 0.068 (.006) | 0.052 (.006) | 0.005 (.002) | 0 (.001) | 0.007 (.002) | 0.002 (.001) | 0.002 (.001) | 0 (0) | 0 (0) | 0 (0) |
|  | 2 (9.62%) | 0.621 (.038) | 0.675 (.045) | 0.251 (.036) | 0.079 (.022) | 0.17 (.024) | 0.074 (.022) | 0.07 (.024) | 0.004 (.003) | 0.002 (.002) | 0.022 (.010) |
|  | 3 (1.44%) | 0.8 (.066) | 0.868 (.068) | 0.864 (.066) | 0.754 (.064) | 0.769 (.095) | 0.847 (.082) | 0.809 (.065) | 0.058 (.032) | 0.241 (.08) | 0.377 (.092) |
| BGPS 2010 | 1 (86.69%) | 0.033 (.004) | 0.112 (.007) | 0.005 (.002) | 0 (0) | 0.005 (.001) | 0.001 (.001) | 0 (0) | 0 (0) | 0 (0) | 0 (0) |
|  | 2 (11.44%) | 0.544 (.038) | 0.776 (.034) | 0.293 (.032) | 0.099 (.022) | 0.158 (.022) | 0.085 (.017) | 0.117 (.02) | 0.001 (.001) | 0.01 (.005) | 0.023 (.008) |
|  | 3 (1.87%) | 0.899 (.068) | 0.975 (.021) | 0.858 (.06) | 0.858 (.051) | 0.807 (.065) | 0.813 (.063) | 0.67 (.076) | 0.158 (.049) | 0.243 (.062) | 0.365 (.071) |
| HSE & SHS 2012 | 1 (90.49%) | 0.057 (.006) | 0.058 (.008) | 0.001 (.001) | 0 (0) | 0.002 (.001) | 0.001 (.001) | 0.001 (.001) | 0 (0) | 0 (0) | 0 (0) |
|  | 2 (8.15%) | 0.618 (.064) | 0.775 (.06) | 0.184 (.041) | 0.035 (.027) | 0.103 (.03) | 0.051 (.024) | 0.066 (.031) | 0.004 (.004) | 0.005 (.011) | 0 (0) |
|  | 3 (1.36%) | 0.795 (.104) | 0.928 (.086) | 0.716 (.109) | 0.798 (.132) | 0.662 (.108) | 0.683 (.123) | 0.794 (.106) | 0.187 (.084) | 0.25 (.085) | 0.339 (.117) |
|  |  |  |  |  |  |  |  |  |  |  |  |
| HSE & SHS 2012 | 1 (88.95%) | 0.053 (.007) | 0.051 (.009) | 0.001 (.001) | 0 (0) | 0.002 (.001) | 0.001 (.001) | 0.001 (.001) | 0 (0) | 0 (0) | 0 (0) |
|  | 2 (9.14%) | 0.558 (.064) | 0.723 (.074) | 0.143 (.033) | 0.007 (.009) | 0.07 (.016) | 0.033 (.015) | 0.038 (.016) | 0.03 (.004) | 0 (0) | 0 (0) |
|  | 3 (1.65%) | 0.754 (.076) | 0.883 (.058) | 0.574 (.075) | 0.631 (.089) | 0.529 (.076) | 0.474 (.08) | 0.613 (.085) | 0.004 (.004) | 0.089 (.039) | 0.118 (.05) |
|  | 4 (0.026%) | 0.775 (.15) | 1 (0) | 1 (0) | 1 (0) | 0.993 (.068) | 1 (0) | 1 (0) | 0.935 (.051) | 0.889 (.107) | 1 (0) |

Table S10. Response probabilities from polytomous LCAs for the DSM-IV Pathological Gambling criteria derived assessment. Groups are ordered by severity/group membership, and standard errors are reported in brackets.

| Item |  |  | 1999 |  |  | 2007 |  |  | 2010 |  |  | 2012 |  |
| --- | --- | --- | --- | --- | --- | --- | --- | --- | --- | --- | --- | --- | --- |
|  |  | L1 (92.10%) | L2 (6.73%) | L3 (1.17%) | L1 (90.15%) | L2 (8.77%) | L3 (1.08%) | L1 (88.19%) | L2 (10.43%) | L3 (1.38%) | L1 (90.78%) | L2 (7.95%) | L3 (1.27%) |
| 1 | 0 | 0.954 (.004) | 0.401 (.042) | 0.126 (.048) | 0.928 (.006) | 0.344 (.034) | 0.153 (.049) | .963 (.005) | .412 (.04) | .021 (.025) | .942 (.006) | .373 (.059) | .182 (.073) |
|  | 1 | 0.034 (.004) | 0.466 (.042) | 0.497 (.079) | 0.031 (.003) | 0.483 (.034) | 0.373 (.081) | .028 (.004) | .464 (.034) | .427 (.082) | .041 (.005) | .523 (.056) | .497 (.085) |
|  | 2 | 0.007 (.001) | 0.085 (.019) | 0.259 (.07) | 0.029 (.003) | 0.148 (.019) | 0.248 (.062) | .006 (.001) | .095 (.019) | .328 (.068) | .009 (.002) | .096 (.023) | .29 (.071) |
|  | 3 | 0.005 (.001) | 0.047 (.015) | 0.118 (.039) | 0.013 (.002) | 0.025 (.011) | 0.226 (.076) | .003 (.001) | .029 (.008) | .224 (.071) | .008 (.002) | .008 (.007) | .031 (.023) |
| 2 | 0 | 0.911 (.006) | 0.21 (.04) | 0.109 (.045) | 0.942 (.005) | 0.3 (.038) | 0.07 (.035) | .881 (.007) | .182 (.032) | .014 (.014) | .94 (.008) | .224 (.065) | .043 (.044) |
|  | 1 | 0.086 (.006) | 0.625 (.035) | 0.388 (.08) | 0.055 (.005) | 0.589 (.034) | 0.279 (.069) | .11 (.007) | .667 (.031) | .279 (.092) | .059 (.007) | .715 (.054) | .522 (.081) |
|  | 2 | 0.002 (.001) | 0.135 (.026) | 0.299 (.072) | 0.003 (.001) | 0.086 (.019) | 0.293 (.079) | .007 (.001) | .12 (.019) | .46 (.073) | .001 (.001) | .043 (.02) | .275 (.066) |
|  | 3 | 0.001 (.001) | 0.03 (.013) | 0.204 (.057) | 0 (0) | 0.024 (.011) | 0.358 (.09) | .002 (.001) | .031 (.009) | .248 (.069) | .001 (.001) | .019 (.009) | .16 (.061) |
| 3 | 0 | 0.996 (.001) | 0.757 (.034) | 0.212 (.064) | 0.994 (.002) | 0.704 (.033) | 0.069 (.044) | .994 (.002) | .66 (.039) | .061 (.037) | .999 (.001) | .814 (.041) | .23 (.81) |
|  | 1 | 0.004 (.001) | 0.22 (.031) | 0.423 (.078) | 0.006 (.002) | 0.28 (.031) | 0.482 (.076) | .005 (.001) | .293 (.033) | .411 (.089) | .001 (.001) | .173 (.039) | .495 (.75) |
|  | 2 | 0 (0) | 0.019 (.009) | 0.259 (.066) | 0 (0) | 0.011 (.007) | 0.296 (.066) | .001 (.001) | .042 (.011) | .341 (.071) | 0 (0) | .011 (.009) | .187 (.74) |
|  | 3 | 0 (0) | 0.003 (.004) | 0.106 (.042) | 0 (0) | 0.004 (.003) | 0.153 (.07) | 0 (0) | .006 (.003) | .187 (.064) | 0 (0) | .002 (.002) | .088 (.44) |
| 4 | 0 | 1 (0) | 0.92 (.019) | 0.233 (.07) | 0.999 (.001) | 0.892 (.023) | 0.182 (.064) | .999 (0) | .851 (.037) | .163 (.057) | 1 (0) | .962 (.03) | .158 (.083) |
|  | 1 | 0 (0) | 0.074 (.018) | 0.42 (.085) | 0.001 (.001) | 0.098 (.02) | 0.285 (.084) | 0 (0) | .115 (.028) | .327 (.092) | 0 (0) | .035 (.029) | .63 (.088) |
|  | 2 | 0 (0) | 0.006 (.004) | 0.207 (.068) | 0 (0) | 0.01 (.006) | 0.344 (.066) | 0 (0) | .033 (.015) | .282 (.062) | 0 (0) | (0) | .133 (.049) |
|  | 3 | 0 (0) | 0 (0) | 0.14 (.048) | 0 (0) | 0 (0) | 0.189 (.056) | .001 (0) | 0 (0) | .228 (.075) | 0 (0) | .003 (.003) | .08 (.039) |
| 5 | 0 | 0.987 (.002) | 0.731 (.033) | 0.204 (.066) | 0.992 (.002) | 0.802 (.026) | 0.148 (.073) | .995 (.001) | .798 (.035) | .166 (.06) | .998 (.001) | .893 (.027) | .302 (.097) |
|  | 1 | 0.012 (.002) | 0.246 (.031) | 0.259 (.076) | 0.008 (.002) | 0.178 (.024) | 0.303 (.077) | .005 (.001) | .188 (.033) | .327 (.089) | .002 (.001) | .098 (.024) | .415 (.072) |
|  | 2 | 0 (0) | 0.017 (.009) | 0.4 (.068) | 0 (0) | 0.011 (.005) | 0.265 (.063) | 0 (0) | .011 (.005) | .247 (.063) | 0 (0) | .005 (.004) | .23 (.065) |
|  | 3 | 0 (0) | 0.006 (.004) | 0.137 (.046) | 0 (0) | 0.01 (.005) | 0.284 (.083) | 0 (0) | .003 (.003) | .26 (.08) | 0 (0) | .004 (.003) | .052 (.032) |

Table S10 (continued)

| Item |  |  | 1999 |  |  | 2007 |  |  | 2010 |  |  | 2012 |  |
| --- | --- | --- | --- | --- | --- | --- | --- | --- | --- | --- | --- | --- | --- |
|  |  | L1 (92.10%) | L2 (6.73%) | L3 (1.17%) | L1 (90.15%) | L2 (8.77%) | L3 (1.08%) | L1 (88.19%) | L2 (10.43%) | L3 (1.38%) | L1 (90.78%) | L2 (7.95%) | L3 (1.27%) |
| 6 | 0 | 1 (0) | 0.937 (.019) | 0.191 (.061) | 0.998 (.001) | 0.885 (.023) | 0.143 (.06) | .999 (.001) | .871 (.03) | .176 (.07) | 0.999 (.001) | 0.938 (.022) | 0.331 (.129) |
|  | 1 | 0 (0) | 0.063 (.019) | 0.427 (.075) | 0.001 (.001) | 0.109 (.022) | 0.371 (.078) | .001 (.001) | .12 (.028) | .467 (.079) | 0.001 (.001) | 0.044 (.016) | 0.329 (.07) |
|  | 2 | 0 (0) | 0 (0) | 0.246 (.064) | 0 (0) | 0.006 (.004) | 0.231 (.048) | 0 (0) | .009 (.005) | .187 (.061) | 0 (0) | 0.013 (.012) | 0.252 (.089) |
|  | 3 | 0 (0) | 0 (0) | 0.136 (.043) | 0 (0) | 0 (0) | 0.256 (.085) | 0 (0) | 0 (0) | .171 (.059) | 0 (0) | 0.005 (.005) | 0.088 (.044) |
| 7 | 0 | 0.999 (.001) | 0.886 (.023) | 0.145 (.061) | 0.998 (.001) | 0.894 (.022) | 0.164 (.064) | .999 (0) | .855 (.026) | .238 (.073) | 0.999 (.001) | 0.922 (.031) | 0.214 (.098) |
|  | 1 | 0.001 (.001) | 0.093 (.02) | 0.405 (.081) | 0 (0) | 0.095 (.021) | 0.452 (.086) | .001 (0) | .104 (.022) | .407 (.07) | 0 (.001) | 0.06 (.023) | 0.428 (.084) |
|  | 2 | 0 (0) | 0.011 (.007) | 0.245 (.063) | 0 (0) | 0.002 (.002) | 0.249 (.073) | 0 (0) | .016 (.007) | .233 (.06) | 0 (0) | 0.015 (.013) | 0.189 (.057) |
|  | 3 | 0 (0) | 0.009 (.006) | 0.205 (.057) | 0.001 (0) | 0.009 (.006) | 0.135 (.051) | 0 (0) | .025 (.007) | .122 (.047) | 0 (0) | 0.003 (.004) | 0.169 (.066) |
| 8 | 0 | 1 (0) | 1 (0) | 0.789 (.063) | 1 (0) | 0.995 (.004) | 0.924 (.041) | 1 (0) | .998 (.002) | .789 (.072) | 1 (0) | 0.996 (.004) | 0.8 (.084) |
|  | 1 | 0 (0) | 0 (0) | 0.089 (.041) | 0 (0) | 0.002 (.002) | 0 (0) | 0 (0) | .002 (.002) | .108 (.044) | 0 (0) | 0 (0) | 0.086 (.046) |
|  | 2 | 0 (0) | 0 (0) | 0.085 (.042) | 0 (0) | 0 (0) | 0.044 (.032) | 0 (0) | 0 (0) | .072 (.037) | 0 (0) | 0 (0) | 0.075 (.044) |
|  | 3 | 0 (0) | 0 (0) | 0.037 (.026) | 0 (0) | 0.003 (.003) | 0.032 (.024) | 0 (0) | 0 (0) | .031 (.023) | 0 (0) | 0.004 (.004) | 0.038 (.029) |
| 9 | 0 | 1 (0) | 0.992 (.005) | 0.745 (.063) | 1 (0) | 0.997 (.003) | 0.692 (.084) | 1 (0) | .992 (.004) | .67 (.094) | 1 (0) | 0.99 (.011) | 0.765 (.088) |
|  | 1 | 0 (0) | 0.008 (.005) | 0.166 (.053) | 0 (0) | 0.002 (.002) | 0.17 (.054) | 0 (0) | .008 (.004) | .187 (.063) | 0 (0) | 0.01 (.011) | 0.143 (.067) |
|  | 2 | 0 (0) | 0 (0) | 0.055 (.032) | 0 (0) | 0 (0) | 0.037 (.026) | 0 (0) | 0 (0) | .075 (.036) | 0 (0) | 0 (0) | 0.047 (.026) |
|  | 3 | 0 (0) | 0 (0) | 0.035 (.025) | 0 (0) | 0.002 (.002) | 0.101 (.047) | 0 (0) | 0 (0) | .068 (.035) | 0 (0) | 0 (0) | 0.045 (.031) |
| 10 | 0 | 1 (0) | 0.986 (.007) | 0.603 (.082) | 1 (0) | 0.971 (.011) | 0.53 (.094) | .999 (0) | .965 (.013) | .601 (.096) | 1 (0) | 0.998 (.002) | 0.651 (.109) |
|  | 1 | 0 (0) | 0.014 (.007) | 0.229 (.061) | 0 (0) | 0.027 (.01) | 0.252 (.06) | 0 (0) | .032 (.013) | .243 (.061) | 0 (0) | 0.002 (.002) | 0.226 (.082) |
|  | 2 | 0 (0) | 0 (0) | 0.13 (.051) | 0 (0) | 0 (0) | 0.126 (.053) | 0 (0) | .004 (.002) | .098 (.045) | 0 (0) | 0 (0) | 0.069 (.031) |
|  | 3 | 0 (0) | 0 (0) | 0.038 (.027) | 0 (0) | 0.002 (.002) | 0.091 (.044) | 0 (0) | 0 (0) | .057 (.033) | 0 (0) | 0 (0) | 0.053 (.034) |

Table S11. Response probabilities for latent classes from Problem Gambling Severity Index data in the BGPS 2007, 2010 and SHS & HSE 2012. Latent classes are ordered by severity/group membership, and standard errors are reported in brackets.

|  |  |  | 2007 | |  | | |  | 2010 | |  | | |  | 2012 |  | |
| --- | --- | --- | --- | --- | --- | --- | --- | --- | --- | --- | --- | --- | --- | --- | --- | --- | --- |
|  |  | L1  (94.84%) | | L2 (4.25%) | | L3 (0.91%) | L1 (93.16%) | | | L2 (5.48%) | | L3 (1.36%) | L1 (96.66%) | | L2 (2.94%) | | L3 (0.4%) |
| 1 | 0 | 0.989 (.002) | | 0.394 (.053) | | 0.093 (.045) | 0.987 (.002) | | | 0.529 (.043) | | 0.18 (.065) | 0.991 (.002) | | 0.538 (.05) | | 0.113 (.067) |
|  | 1 | 0.01 (.002) | | 0.592 (.053) | | 0.464 (.114) | 0.012 (.002) | | | 0.445 (.041) | | 0.382 (.081) | 0.009 (.002) | | 0.426 (.05) | | 0.353 (.097) |
|  | 2 | 0 (0) | | 0.013 (.008) | | 0.232 (.063) | 0 (0) | | | 0.022 (.01) | | 0.225 (.058) | 0 (0) | | 0.03 (.016) | | 0.229 (.102) |
|  | 3 | 0 (0) | | 0 (0) | | 0.211 (.081) | 0 (0) | | | 0.004 (.004) | | 0.213 (.069) | 0 (0) | | 0.006 (.009) | | 0.305 (.104) |
| 2 | 0 | 0.997 (.001) | | 0.753 (.046) | | 0.254 (.071) | 0.999 (.001) | | | 0.795 (.03) | | 0.452 (.08) | 0.998 (.001) | | 0.771 (.044) | | 0.245 (.115) |
|  | 1 | 0.003 (.001) | | 0.241 (.046) | | 0.405 (.097) | 0.001 (.001) | | | 0.196 (.028) | | 0.358 (.074) | 0.002 (.001) | | 0.226 (.044) | | 0.49 (.116) |
|  | 2 | 0 (0) | | 0.006 (.006) | | 0.225 (.087) | 0 (0) | | | 0.009 (.007) | | 0.096 (.034) | 0 (0) | | 0.001 (.001) | | 0.077 (.045) |
|  | 3 | 0 (0) | | 0 (0) | | 0.115 (.05) | 0 (0) | | | 0 (0) | | 0.095 (.045) | 0 (0) | | 0.002 (.002) | | 0.188 (.1) |
| 3 | 0 | 0.963 (.004) | | 0.326 (.045) | | 0.087 (.046) | 0.978 (.003) | | | 0.43 (.045) | | 0.135 (.061) | 0.975 (.003) | | 0.388 (.049) | | 0.143 (.075) |
|  | 1 | 0.033 (.004) | | 0.644 (.045) | | 0.412 (.107) | 0.021 (.003) | | | 0.539 (.045) | | 0.478 (.075) | 0.022 (.003) | | 0.563 (.051) | | 0.462 (.122) |
|  | 2 | 0.002 (.001) | | 0.016 (.014) | | 0.293 (.07) | 0 (0) | | | 0.031 (.012) | | 0.214 (.056) | 0.002 (.001) | | 0.048 (.022) | | 0.241 (.092) |
|  | 3 | 0.002 (.001) | | 0.014 (.008) | | 0.208 (.087) | 0.001 (0) | | | 0 (0) | | 0.173 (.057) | 0.001 (0) | | 0.001 (.001) | | 0.154 (.087) |
| 4 | 0 | 0.999 (0) | | 0.901 (.025) | | 0.376 (.106) | 1 (0) | | | 0.927 (.023) | | 0.321 (.07) | 1 (0) | | 0.921 (.026) | | 0.302 (.106) |
|  | 1 | 0.001 (0) | | 0.095 (.025) | | 0.475 (.085) | 0 (0) | | | 0.069 (.022) | | 0.539 (.071) | 0 (0) | | 0.079 (.026) | | 0.439 (.097) |
|  | 2 | 0 (0) | | 0 (0) | | 0.062 (.035) | 0 (0) | | | 0.005 (.005) | | 0.053 (.026) | 0 (0) | | 0 (0) | | 0.122 (.068) |
|  | 3 | 0 (0) | | 0.004 (.004) | | 0.087 (.046) | 0 (0) | | | 0 (0) | | 0.086 (.038) | 0 (0) | | 0 (0) | | 0.137 (.086) |
| 5 | 0 | 1 (0) | | 0.855 (.035) | | 0.095 (.052) | 0.999 (0) | | | 0.873 (.035) | | 0.07 (.039) | 1 (.001) | | 0.65 (.059) | | 0 (0) |
|  | 1 | 0 (0) | | 0.145 (.035) | | 0.444 (.105) | 0.001 (0) | | | 0.127 (.035) | | 0.598 (.075) | 0 (.001) | | 0.332 (.058) | | 0.424 (.139) |
|  | 2 | 0 (0) | | 0 (0) | | 0.178 (.066) | 0 (0) | | | 0 (0) | | 0.154 (.053) | 0 (0) | | 0.015 (.01) | | 0.302 (.092) |
|  | 3 | 0 (0) | | 0 (0) | | 0.282 (.111) | 0 (0) | | | 0 (0) | | 0.164 (.063) | 0 (0) | | 0.002 (.002) | | 0.274 (.106) |

Table S11 (continued)

|  |  | L1  (94.84%) | L2 (4.25%) | L3 (0.91%) | L1 (93.16%) | L2 (5.48%) | L3 (1.36%) | L1 (96.66%) | L2 (2.96%) | L3 (0.37%) |
| --- | --- | --- | --- | --- | --- | --- | --- | --- | --- | --- |
| 6 | 0 | 1 (0) | 0.934 (.024) | 0.254 (.091) | 0.999 (.001) | 0.92 (.022) | 0.167 (.062) | 1 (0) | 0.756 (.05) | 0.065 (.054) |
|  | 1 | 0 (0) | 0.062 (.023) | 0.466 (.085) | 0.001 (.001) | 0.067 (.021) | 0.642 (.065) | 0 (0) | 0.237 (.05) | 0.317 (.126) |
|  | 2 | 0 (0) | 0.005 (.005) | 0.096 (.048) | 0 (0) | 0.009 (.005) | 0.078 (.033) | 0 (0) | 0.006 (.006) | 0.365 (.112) |
|  | 3 | 0 (0) | 0 (0) | 0.184 (.081) | 0 (0) | 0.004 (.004) | 0.113 (.047) | 0 (0) | 0 (0) | 0.253 (.102) |
| 7 | 0 | 0.995 (.001) | 0.714 (.044) | 0.176 (.074) | 0.996 (.001) | 0.712 (.051) | 0.283 (.066) | 0.997 (.001) | 0.66 (.055) | 0.018 (.049) |
|  | 1 | 0.004 (.001) | 0.27 (.043) | 0.416 (.073) | 0.004 (.001) | 0.264 (.048) | 0.409 (.075) | 0.002 (.001) | 0.296 (.053) | 0.544 (.11) |
|  | 2 | 0 (0) | 0.015 (.01) | 0.163 (.062) | 0 (0) | 0.025 (.012) | 0.144 (.048) | 0 (0) | 0.031 (.015) | 0.114 (.061) |
|  | 3 | 0 (0) | 0 (0) | 0.245 (.087) | 0 (0) | 0 (0) | 0.164 (.054) | 0 (0) | 0.013 (.013) | 0.323 (.114) |
| 8 | 0 | 1 (0) | 0.891 (.035) | 0.121 (.058) | 0.999 (.001) | 0.934 (.021) | 0.187 (.073) | 1 (0) | 0.867 (.039) | 0.011 (.011) |
|  | 1 | 0 (0) | 0.1 (.031) | 0.503 (.099) | 0.001 (.001) | 0.063 (.02) | 0.562 (.071) | 0 (0) | 0.121 (.034) | 0.543 (.106) |
|  | 2 | 0 (0) | 0.09 (.011) | 0.124 (.05) | 0 (0) | 0.003 (.003) | 0.061 (.026) | 0 (0) | 0.013 (.013) | 0.191 (.081) |
|  | 3 | 0 (0) | 0 (0) | 0.252 (.111) | 0 (0) | 0 (0) | 0.19 (.054) | 0 (0) | 0 (0) | 0.256 (.102) |
| 9 | 0 | 0.996 (.001) | 0.721 (.045) | 0.061 (.041) | 0.995 (.001) | 0.645 (.051) | 0.199 (.055) | 0.999 (.001) | 0.492 (.066) | 0.053 (.052) |
|  | 1 | 0.003 (.001) | 0.269 (.045) | 0.461 (.115) | 0.005 (.001) | 0.341 (.047) | 0.5 (.076) | 0.001 (.001) | 0.485 (.064) | 0.407 (.14) |
|  | 2 | 0 (0) | 0.005 (.005) | 0.17 | 0.001 (0) | 0.012 (.008) | 0.108 (.036) | 0 (0) | 0.023 (.015) | 0.222 (.088) |
|  | 3 | 0 (0) | 0.004 (.004) | 0.308 | 0 (0) | 0.002 (.006) | 0.193 (.053) | 0 (0) | 0 (0) | 0.318 (.121) |

Table S12. Response probabilities for the LCA conducted on a DSM-IV Pathological Gambling criteria derived assessment administered in the APMS 2007. Classes are ordered by severity/group membership, and standard errors are reported in brackets.

| Item | L1 – 97.58% | L2 – 2.02% | L3 - 0.41% |
| --- | --- | --- | --- |
| 1 | 0.004 (.002) | 0.374 (.067) | 0.975 (.038) |
| 2 | 0.001 (.001) | 0.188 (.051) | 0.947 (.07) |
| 3 | 0.009 (.002) | 0.285 (.066) | 1 (0) |
| 4 | 0 (0) | 0.071 (.032) | 1 (0) |
| 5 | 0.003 (.001) | 0.274 (.068) | 1 (0) |
| 6 | 0.006 (.002) | 0.437 (.075) | 1 (0) |
| 7 | 0.001 (.001) | 0.086 (.042) | 0.922 (.072) |
| 8 | 0 (0) | 0.035 (.018) | 0.379 (.168) |
| 9 | 0.001 (.001) | 0.051 (.023) | 0.616 (.16) |
| 10 | 0 (0) | 0.04 (.026) | 0.613 (.17) |

Note: The order in which these were presented to respondents differed from the other DSM measure (see Table S1). The order used in the graphs to compare these is as follows: 6, 1, 2, 4, 5, 7, 3, 8, 9, 10.

Table S13. Response probabilities for the LCA conducted on the South Oaks Gambling Screen Data collected as part of the BGPS 1999 questionnaire. Groups are ordered by severity/group membership, and standard errors are reported in brackets.

|  | L1 – 93.72% | L2 – 5.39% | L3 – 0.9% |
| --- | --- | --- | --- |
| 4 | 0.015 (.002) | 0.124 (.026) | 0.52 (.099) |
| 5 | 0.011 (.002) | 0.224 (.045) | 0.601 (.081) |
| 7 | 0.019 (.003) | 0.412 (.058) | 0.736 (.08) |
| 8 | 0.014 (.003) | 0.475 (.059) | 0.704 (.075) |
| 9 | 0.006 (.002) | 0.297 (.052) | 0.76 (.074) |
| 10 | 0.005 (.001) | 0.093 (.024) | 0.684 (.099) |
| 11 | 0.004 (.001) | 0.104 (.02) | 0.482 (.098) |
| 13 | 0 (0) | 0.079 (.025) | 0.462 (.089) |
| 15 | 0 (0) | 0.025 (.013) | 0.149 (.057) |
| 14 | 0 (0) | 0.033 (.015) | 0.358 (.089) |
| 16a | 0.001 (.001) | 0.089 (.03) | 0.442 (.09) |
| 16b | 0.003 (.001) | 0.147 (.025) | 0.303 (.077) |
| 16c | 0 (0) | 0.072 (.028) | 0.328 (.079) |
| 16d | 0 (0) | 0.011 (.007) | 0.2 (.072) |
| 16e | 0.002 (.001) | 0.066 (.019) | 0.467 (.108) |
| 16f | 0 (0) | 0 (0) | 0.083 (.045) |
| 16g | 0 (0) | 0.007 (.005) | 0.061 (.038) |
| 16h | 0 (0) | 0 (0) | 0.169 (.075) |
| 16i | 0 (0) | 0 (0) | 0.229 (.074) |
| 6 | 0 (0) | 0.016 (.012) | 0.386 (.087) |

**Note: The order in which the SOGS questions were administered was slightly different to the screen as reported in the SOGS (**[**28**](#_ENREF_28)**). The order in this table represents the order respondents in the BGPS were given the questions.**

Table S14. Means item scores for each of the Problem Gambling Severity Index items, for each of the three latent classes from each of the surveys that the PGSI was administered in (BGPS 2007, BGPS 2010, SHS & HSE 2012).

|  |  | 1 | 2 | 3 | 4 | 5 | 6 | 7 | 8 | 9 |
| --- | --- | --- | --- | --- | --- | --- | --- | --- | --- | --- |
| BGPS 2007 | 1 | 0.0131 | 0.0032 | 0.0446 | 0.0007 | 0.0006 | 0.0003 | 0.0062 | 0 | 0.0041 |
|  | 2 | 0.6969 | 0.2983 | 0.8043 | 0.1175 | 0.1689 | 0.0791 | 0.3553 | 0.1416 | 0.3342 |
|  | 3 | 1.5652 | 1.2027 | 1.6337 | 0.8820 | 1.6438 | 1.2244 | 1.4626 | 1.5076 | 1.7375 |
| BGPS 2010 | 1 | 0.0152 | 0.0021 | 0.0276 | 0.0012 | 0.0014 | 0.0012 | 0.0058 | 0.0010 | 0.0081 |
|  | 2 | 0.6158 | 0.2659 | 0.7109 | 0.1034 | 0.1563 | 0.1267 | 0.3917 | 0.0842 | 0.4456 |
|  | 3 | 1.4736 | 0.8439 | 1.4163 | 0.9025 | 1.4429 | 1.1293 | 1.1756 | 1.2614 | 1.3240 |
| SHS/HSE  2012 | 1 | 0.0096 | 0.0024 | 0.0291 | 0.0004 | 0 | 0.0005 | 0.0029 | 0 | 0.0017 |
|  | 2 | 0.5550 | 0.2481 | 0.7146 | 0.0815 | 0.4050 | 0.2636 | 0.4211 | 0.1607 | 0.5522 |
|  | 3 | 1.7335 | 1.2339 | 1.4257 | 1.1319 | 1.8701 | 1.8312 | 1.7721 | 1.7021 | 1.8237 |

Table S15. Mean items scores for each of the adapted DSM-IV Pathological Gambling criteria assessment items, for each of the three latent classes from each of the surveys that the PGSI was administered in (BGPS 1999, BGPS 2007, BGPS 2010, SHS & HSE 2012).

|  |  | 1 | 2 | 3 | 4 | 5 | 6 | 7 | 8 | 9 | 10 |
| --- | --- | --- | --- | --- | --- | --- | --- | --- | --- | --- | --- |
| BGPS 1999 | 1 | 0.0971 | 0.0048 | 0 | 0.0111 | 0.0010 | 0.0593 | 0.0004 | 0.0004 | 0 | 0 |
|  | 2 | 1.0574 | 0.3100 | 0.0959 | 0.3648 | 0.1653 | 0.9493 | 0.0707 | 0 | 0.0099 | 0.0151 |
|  | 3 | 1.6031 | 1.2587 | 1.2725 | 1.4886 | 1.5060 | 1.3704 | 1.3304 | 0.3717 | 0.3796 | 0.6084 |
| BGPS 2007 | 1 | 0.1271 | 0.0633 | 0.0065 | 0.0010 | 0.0075 | 0.0022 | 0.0025 | 0 | 0.0009 | 0.0009 |
|  | 2 | 0.9833 | 0.9599 | 0.3682 | 0.1369 | 0.2821 | 0.1415 | 0.1645 | 0.0124 | 0.0080 | 0.0372 |
|  | 3 | 1.5890 | 1.9459 | 1.5509 | 1.5849 | 1.7206 | 1.6390 | 1.3898 | 0.1893 | 0.5646 | 0.8043 |
| BGPS 2010 | 1 | 0.0460 | 0.1352 | 0.0075 | 0.0017 | 0.0047 | 0.0017 | 0.0012 | 0.0002 | 0.0009 | 0.0013 |
|  | 2 | 0.8715 | 1.0656 | 0.4439 | 0.2131 | 0.2498 | 0.1504 | 0.2361 | 0.0015 | 0.0095 | 0.0432 |
|  | 3 | 1.7750 | 1.9580 | 1.6653 | 1.5859 | 1.6560 | 1.4124 | 1.2564 | 0.3594 | 0.5610 | 0.6331 |
| SHS/HSE 2012 | 1 | 0.0861 | 0.0736 | 0.0021 | 0 | 0.0024 | 0.0017 | 0.0008 | 0 | 0 | 0.0002 |
|  | 2 | 0.9367 | 0.9833 | 0.2639 | 0.0628 | 0.1679 | 0.1130 | 0.1468 | 0.0162 | 0.0164 | 0.0036 |
|  | 3 | 1.1834 | 1.5587 | 1.1855 | 1.1705 | 1.0489 | 1.1363 | 1.3398 | 0.3667 | 0.3788 | 0.5447 |

Table S16. Frequency distributions for each score compared against each latent class on the assessment derived from the DSM-IV Pathological Gambling criteria, scored using the same method as the BGPS reports (items rated from 0-3 by respondent, scored as present on items 1 – 7 if > 1, on items 8 – 10 if > 0).

|  | BGPS 1999 | | | BGPS 2007 | | | BGPS 2010 | | | SHS/HSE 2012 | | |
| --- | --- | --- | --- | --- | --- | --- | --- | --- | --- | --- | --- | --- |
| DSM Score | 1 | 2 | 3 | 1 | 2 | 3 | 1 | 2 | 3 | 1 | 2 | 3 |
| 0 | 5005 | 0 | 0 | 5065 | 0 | 0 | 5310 | 0 | 0 | 6592 | 0 | 0 |
| 1 | 168 | 9 | 0 | 346 | 5 | 0 | 239 | 18 | 0 | 238 | 1 | 0 |
| 2 | 0 | 35 | 0 | 2 | 33 | 0 | 0 | 60 | 0 | 0 | 36 | 0 |
| 3 | 0 | 8 | 2 | 0 | 17 | 2 | 0 | 24 | 0 | 0 | 14 | 1 |
| 4 | 0 | 5 | 6 | 0 | 2 | 4 | 0 | 13 | 1 | 0 | 6 | 3 |
| 5 | 0 | 0 | 2 | 0 | 2 | 7 | 0 | 2 | 7 | 0 | 0 | 0 |
| 6 | 0 | 0 | 12 | 0 | 0 | 6 | 0 | 0 | 7 | 0 | 3 | 4 |
| 7 | 0 | 0 | 2 | 0 | 0 | 1 | 0 | 0 | 8 | 0 | 2 | 1 |
| 8 | 0 | 0 | 2 | 0 | 0 | 7 | 0 | 0 | 6 | 0 | 0 | 1 |
| 9 | 0 | 0 | 1 | 0 | 0 | 1 | 0 | 0 | 2 | 0 | 0 | 3 |
| 10 | 0 | 0 | 1 | 0 | 0 | 2 | 0 | 0 | 2 | 0 | 0 | 2 |

Table S17. Frequency distributions for each score compared against each latent class on the assessment derived from the DSM-IV Pathological Gambling criteria, scored as a symptom being present if any response other than ‘Never’ was given.

|  | BGPS 1999 | | | BGPS 2007 | | | BGPS 2010 | | | SHS/HSE 2012 | | |
| --- | --- | --- | --- | --- | --- | --- | --- | --- | --- | --- | --- | --- |
| DSM Score | 1 | 2 | 3 | 1 | 2 | 3 | 1 | 2 | 3 | 1 | 2 | 3 |
| 0 | 4151 | 0 | 0 | 4286 | 0 | 0 | 4222 | 0 | 0 | 5554 | 0 | 0 |
| 1 | 738 | 0 | 0 | 744 | 5 | 0 | 861 | 10 | 0 | 854 | 14 | 0 |
| 2 | 0 | 198 | 0 | 0 | 246 | 0 | 0 | 308 | 0 | 0 | 286 | 0 |
| 3 | 0 | 68 | 0 | 0 | 107 | 0 | 0 | 118 | 0 | 0 | 85 | 5 |
| 4 | 0 | 34 | 0 | 0 | 33 | 5 | 0 | 64 | 0 | 0 | 23 | 11 |
| 5 | 0 | 17 | 7 | 0 | 4 | 18 | 0 | 8 | 30 | 0 | 0 | 27 |
| 6 | 0 | 0 | 12 | 0 | 0 | 19 | 0 | 0 | 26 | 0 | 0 | 20 |
| 7 | 0 | 0 | 15 | 0 | 0 | 15 | 0 | 0 | 26 | 0 | 0 | 13 |
| 8 | 0 | 0 | 5 | 0 | 0 | 9 | 0 | 0 | 11 | 0 | 0 | 3 |
| 9 | 0 | 0 | 8 | 0 | 0 | 7 | 0 | 0 | 5 | 0 | 0 | 6 |
| 10 | 0 | 0 | 4 | 0 | 0 | 3 | 0 | 0 | 10 | 0 | 0 | 11 |

Table S18. Frequency distributions for each score compared against each latent class for the Problem Gambling Severity Index.

|  | BGPS 2007 | | | BGPS 2010 | | | SHS/HSE 2012 | | |
| --- | --- | --- | --- | --- | --- | --- | --- | --- | --- |
| PGSI Score | 1 | 2 | 3 | 1 | 2 | 3 | 1 | 2 | 3 |
| 0 | 4984 | 0 | 0 | 5075 | 0 | 0 | 6453 | 0 | 0 |
| 1 | 312 | 1 | 0 | 300 | 124 | 0 | 254 | 4 | 0 |
| 2 | 15 | 99 | 0 | 9 | 47 | 0 | 18 | 77 | 0 |
| 3 | 15 | 47 | 0 | 9 | 28 | 0 | 8 | 45 | 0 |
| 4 | 0 | 23 | 0 | 0 | 30 | 1 | 0 | 19 | 0 |
| 5 | 0 | 17 | 0 | 0 | 5 | 0 | 0 | 19 | 0 |
| 6 | 0 | 7 | 0 | 0 | 0 | 7 | 0 | 19 | 0 |
| 7 | 0 | 5 | 6 | 0 | 0 | 12 | 0 | 4 | 0 |
| 8 | 0 | 0 | 7 | 0 | 0 | 14 | 0 | 4 | 0 |
| 9 | 0 | 0 | 13 | 0 | 0 | 15 | 0 | 2 | 3 |
| 10 | 0 | 0 | 2 | 0 | 0 | 4 | 0 | 0 | 2 |
| 11 | 0 | 0 | 1 | 0 | 0 | 3 | 0 | 0 | 6 |
| 12 | 0 | 0 | 2 | 0 | 0 | 1 | 0 | 0 | 3 |
| 13 | 0 | 0 | 1 | 0 | 0 | 4 | 0 | 0 | 2 |
| 14 | 0 | 0 | 1 | 0 | 0 | 0 | 0 | 0 | 3 |
| 15 | 0 | 0 | 1 | 0 | 0 | 0 | 0 | 0 | 4 |
| 16 | 0 | 0 | 4 | 0 | 0 | 1 | 0 | 0 | 0 |
| 17 | 0 | 0 | 1 | 0 | 0 | 4 | 0 | 0 | 0 |
| 18 | 0 | 0 | 0 | 0 | 0 | 1 | 0 | 0 | 1 |
| 19 | 0 | 0 | 3 | 0 | 0 | 3 | 0 | 0 | 0 |
| 20 | 0 | 0 | 1 | 0 | 0 | 0 | 0 | 0 | 0 |
| 21 | 0 | 0 | 1 | 0 | 0 | 2 | 0 | 0 | 0 |
| 22 | 0 | 0 | 0 | 0 | 0 | 0 | 0 | 0 | 1 |
| 23 | 0 | 0 | 2 | 0 | 0 | 1 | 0 | 0 | 0 |
| 24 | 0 | 0 | 0 | 0 | 0 | 1 | 0 | 0 | 0 |
| 25 | 0 | 0 | 0 | 0 | 0 | 1 | 0 | 0 | 0 |
| 26 | 0 | 0 | 0 | 0 | 0 | 1 | 0 | 0 | 0 |
| 27 | 0 | 0 | 4 | 0 | 0 | 1 | 0 | 0 | 4 |

Table S19. Frequency distributions compared for each score against each latent class for the South Oaks Gambling Screen data from the BGPS 1999.

| SOGS Score | 1 | 2 | 3 |
| --- | --- | --- | --- |
| 0 | 4345 | 0 | 0 |
| 1 | 408 | 1 | 0 |
| 2 | 5 | 102 | 0 |
| 3 | 0 | 60 | 0 |
| 4 | 0 | 27 | 0 |
| 5 | 0 | 12 | 3 |
| 6 | 0 | 6 | 6 |
| 7 | 0 | 0 | 9 |
| 8 | 0 | 0 | 13 |
| 9 | 0 | 0 | 3 |
| 10 | 0 | 0 | 2 |
| 11 | 0 | 0 | 0 |
| 12 | 0 | 0 | 2 |
| 13 | 0 | 0 | 1 |
| 14 | 0 | 0 | 1 |
| 15 | 0 | 0 | 0 |
| 16 | 0 | 0 | 0 |
| 17 | 0 | 0 | 1 |
| 18 | 0 | 0 | 1 |
| 19 | 0 | 0 | 0 |
| 20 | 0 | 0 | 0 |

Table S20. Frequency distributions compared for each scored against each latent class for the DSM-IV Pathological Gambling criteria-derived assessment used in the APMS 2007.

| DSM Score | 1 | 2 | 3 |
| --- | --- | --- | --- |
| 0 | 3362 | 0 | 0 |
| 1 | 120 | 1 | 0 |
| 2 | 0 | 43 | 0 |
| 3 | 0 | 22 | 0 |
| 4 | 0 | 1 | 0 |
| 5 | 0 | 5 | 0 |
| 6 | 0 | 0 | 1 |
| 7 | 0 | 0 | 5 |
| 8 | 0 | 0 | 0 |
| 9 | 0 | 0 | 3 |
| 10 | 0 | 0 | 5 |

Table S21. Demographic and gambling behaviour variables for each latent class for the BGPS 1999 DSM >0 LCA.

|  | Class 1 (*S.D.*) | Class 2 (*S.D*) | Class 3 (*S.D.*) |
| --- | --- | --- | --- |
| Age | 45.23 (17.49) | 36.4 (15.07) | 30.7 (12.87) |
| Number of gambling activities (past year) | 2.12 (1.36) | 3.72 (1.99) | 4.71 (2.75) |
| Past week spend (£): |  |  |  |
| National Lottery | 1.77 (2.7) | 2.71 (3.99) | 3.92 (7.6) |
| Other lottery | 0.114 (0.64) | 0.456 (1.82) | 1.026 (4.87) |
| Pools | 0.192 (0.972) | 0.994 (4.76) | 0.682 (1.65) |
| Bingo | 0.287 (1.91) | 1.278 (5.09) | 1.19 (7.14) |
| Number of gambling activities (past week) | 1.1 (0.97) | 2.27 (1.69) | 2.8 (1.84) |
| Attitudes toward gambling score | 15.26 (6.85) | 21.32 (5.67) | 21.45 (5.5) |
| Sex (REF: Male) | 0.503 | 0.712 | 0.733 |
| *Marital Status:* |  |  |  |
| Married | 0.662 | 0.459 | 0.34 |
| Separated/Divorced | 0.073 | 0.108 | 0.034 |
| Single | 0.18 | 0.376 | 0.605 |
| Widowed | 0.07 | 0.034 | 0 |
| Proportion of class members played: |  |  |  |
| National Lottery | 0.906 | 0.904 | 0.835 |
| Other Lottery | 0.104 | 0.212 | 0.344 |
| Scratchcards | 0.291 | 0.545 | 0.571 |
| Pools | 0.117 | 0.218 | 0.222 |
| Bingo | 0.099 | 0.14 | 0.27 |
| Slots | 0.178 | 0.442 | 0.659 |
| Private Betting | 0.147 | 0.36 | 0.429 |
| Horse Racing | 0.17 | 0.4 | 0.487 |
| Dog Racing | 0.045 | 0.2 | 0.27 |
| Other Betting | 0.03 | 0.178 | 0.359 |
| Casino Games | 0.03 | 0.109 | 0.268 |
| Other Gambling | 0.002 | 0.011 | 0 |

Table S22. Demographic and gambling behaviour variables for each latent class for the BGPS 1999 SOGS LCA.

|  | Class 1 (*S.D.*) | Class 2 (*S.D*) | Class 3 (*S.D.*) |
| --- | --- | --- | --- |
| Age | 44.84 (17.48) | 35.69 (15.05) | 32.98 (13.51) |
| Number of gambling activities (past year) | 2.15 (1.4) | 4.1 (1.97) | 4.84 (2.19) |
| Past week spend (£): |  |  |  |
| National Lottery | 1.79 (2.72) | 3.13 (6.16) | 4.12 (6.72) |
| Other lottery | 0.12 (0.71) | 0.73 (3.64) | 0.578 (1.75) |
| Pools | 0.19 (0.97) | 1.22 (5.68) | 1.3 (3.49) |
| Bingo | 0.3 (1.96) | 1.57 (6.66) | 1.66 (5.73) |
| Number of gambling activities (past week) | 1.13 (1.00) | 2.24 (1.81) | 3.35 (1.99) |
| Attitudes toward gambling score | 15.48 (6.83) | 21.52 (5.07) | 21.57 (6) |
| Sex (REF: Male) | 0.506 | 0.757 | 0.72 |
| *Marital Status:* |  |  |  |
| Married | 0.654 | 0.457 | 0.405 |
| Separated/Divorced | 0.074 | 0.066 | 0.132 |
| Single | 0.188 | 0.42 | 0.442 |
| Widowed | 0.069 | 0.031 | 0.021 |
| Proportion of class members played: |  |  |  |
| National Lottery | 0.906 | 0.888 | 0.881 |
| Other Lottery | 0.106 | 0.258 | 0.221 |
| Scratchcards | 0.299 | 0.569 | 0.507 |
| Pools | 0.118 | 0.233 | 0.27 |
| Bingo | 0.099 | 0.166 | 0.302 |
| Slots | 0.184 | 0.533 | 0.656 |
| Private Betting | 0.15 | 0.446 | 0.557 |
| Horse Racing | 0.172 | 0.479 | 0.537 |
| Dog Racing | 0.049 | 0.211 | 0.285 |
| Other Betting | 0.034 | 0.161 | 0.36 |
| Casino Games | 0.032 | 0.15 | 0.261 |
| Other Gambling | 0.002 | 0.006 | 0 |

Table S23. Differences in demographics and gambling engagement between latent class for the BGPS 2007 DSM >0 cutoff LCA.

|  | Class 1 (*S.D.*) | Class 2 (*S.D*) | Class 3 (*S.D.*) |
| --- | --- | --- | --- |
| Age | 47.02 (17.33) | 36.63 (15.47) | 36.63 (12.6) |
| Number of gambling activities (past year) | 2.32 (1.6) | 4.63 (2.71) | 5.99 (3.69) |
| Most units drank in one day (past week) | 5.84 (6.42) | 9.2 (8.25) | 16.25 (17.18) |
| General health | 1.91 (0.821) | 2.05 (0.839) | 2.21 (0.857) |
| Age of first gamble | 21.02 (10.47) | 17.45 (5.73) | 17.73 (6.72) |
| Number of gambling activities (past week) | 0.88 (0.9) | 1.74 (1.8) | 2.87 (2.47) |
| Sex (REF: Male) | 0.487 | 0.702 | 0.783 |
| *Marital Status:* |  |  |  |
| Married | 0.574 | 0.436 | 0.278 |
| Separated/Divorced | 0.081 | 0.009 | 0.115 |
| Single | 0.249 | 0.422 | 0.542 |
| Widowed | 0.067 | 0.026 | 0.005 |
| Smoking status (REF: Y) | 0.254 | 0.429 | 0.526 |
| Drinking status (REF: Y) | 0.783 | 0.84 | 0.7 |
| Proportion of class members played: |  |  |  |
| National Lottery | 0.872 | 0.818 | 0.916 |
| Scratchcards | 0.274 | 0.538 | 0.539 |
| Other Lottery | 0.169 | 0.225 | 0.277 |
| Pools | 0.043 | 0.118 | 0.089 |
| Bingo | 0.101 | 0.176 | 0.355 |
| Slot Machines | 0.186 | 0.521 | 0.634 |
| FOBT in bookmaker | 0.023 | 0.161 | 0.421 |
| Casino games | 0.046 | 0.19 | 0.275 |
| Online casino games | 0.023 | 0.213 | 0.261 |
| Online betting | 0.038 | 0.179 | 0.227 |
| Betting exchange | 0.009 | 0.072 | 0.103 |
| Horse racing in person | 0.245 | 0.451 | 0.54 |
| Dog racing in person | 0.063 | 0.193 | 0.402 |
| Other betting at bookmaker | 0.064 | 0.26 | 0.318 |
| Spread betting | 0.005 | 0.055 | 0.09 |
| Private betting | 0.133 | 0.394 | 0.425 |
| Other gambling | 0.006 | 0.019 | 0.032 |

Table S24. Demographic and gambling behaviour variables for each latent class for the BGPS 2010 DSM > 0 LCA.

|  | Class 1 (*S.D.*) | Class 2 (*S.D.*) | Class 3 (*S.D.*) |
| --- | --- | --- | --- |
| Age | 47.6 (18.14) | 36.18 (15.1) | 34.24 (13.24) |
| Number of gambling activities (past year) | 2.4 (1.7) | 4.58 (2.92) | 6.21 (3.09) |
| Most units drank in one day (past week frame) | 5.4 (6.79) | 7.07 (7.73) | 10.04 (14.96) |
| General Health | 1.96 (0.88) | 1.94 (0.84) | 2.09 (0.98) |
| Age of first gamble | 19.33 (10.88) | 16.74 (6.264) | 17.71 (6.4) |
| Estimated monthly gambling spend | 15.86 (42.35) | 86.77 (215.69) | 211.958 (444.66) |
| Sex (REF: Male) | 0.483 | 0.667 | 0.81 |
| *Marital Status:* |  |  |  |
| Married | 0.641 | 0.511 | 0.461 |
| Separated/Divorced | 0.085 | 0.065 | 0.077 |
| Single | 0.209 | 0.401 | 0.455 |
| Widowed | 0.065 | 0.022 | 0.007 |
| Smoker (REF: Yes) | 0.255 | 0.401 | 0.528 |
| Drinker (REF: Yes) | 0.78 | 0.782 | 0.778 |
| *Proportion played:* |  |  |  |
| National Lottery | 0.805 | 0.796 | 0.778 |
| Scratchcard | 0.309 | 0.54 | 0.616 |
| Other Lottery | 0.344 | 0.32 | 0.403 |
| Bingo (Online + Land) | 0.106 | 0.244 | 0.218 |
| Pools | 0.046 | 0.16 | 0.28 |
| Slot Machines | 0.144 | 0.423 | 0.487 |
| EGM at bookmaker | 0.032 | 0.251 | 0.414 |
| Poker | 0.016 | 0.101 | 0.217 |
| Casino games (Online + Land) | 0.049 | 0.253 | 0.384 |
| Online fruit/slot machine/ instant win | 0.022 | 0.172 | 0.205 |
| Horse racing | 0.205 | 0.349 | 0.431 |
| Dog racing | 0.048 | 0.141 | 0.281 |
| Spread betting | 0.008 | 0.055 | 0.115 |
| Private betting | 0.136 | 0.297 | 0.501 |
| Other sports bets | 0.091 | 0.318 | 0.508 |
| Other bets | 0.004 | 0.163 | 0.366 |
| Bingo in person | 0.094 | 0.18 | 0.21 |
| Bingo online | 0.017 | 0.091 | 0.041 |
| Slots (inc FOBT prompt) | 0.146 | 0.433 | 0.5 |
| FOBT | 0.028 | 0.212 | 0.332 |
| Casino person | 0.038 | 0.159 | 0.3 |
| Casino online | 0.014 | 0.15 | 0.128 |
| Online gaming | 0.044 | 0.287 | 0.321 |
| Horse in person | 0.19 | 0.299 | 0.409 |
| Horse online | 0.022 | 0.071 | 0.064 |
| Dogs in person | 0.047 | 0.129 | 0.249 |
| Dogs online | 0.001 | 0.022 | 0.041 |
| Sports/other betting land | 0.092 | 0.298 | 0.569 |
| Other betting in person | 0.007 | 0.052 | 0.075 |
| Sports online | 0.023 | 0.121 | 0.093 |
| Sports in person | 0.075 | 0.254 | 0.486 |
| Online betting exchange | 0.008 | 0.044 | 0.046 |
| Online bookmaker | 0.031 | 0.129 | 0.117 |
| Online bookmaker + exchange | 0.036 | 0.151 | 0.139 |
| Online gaming + lottery | 0.154 | 0.368 | 0.359 |
| Online gaming | 0.048 | 0.307 | 0.349 |

Please see note below

Table S25. Demographic and gambling behaviour variables for each latent class for the BGPS 2010 DSM > 0 LCA.

|  | Class 1 (*S.D.*) | Class 2 (*S.D.*) | Class 3 (*S.D.*) |
| --- | --- | --- | --- |
| Age | 47.07 (18.14) | 33.8 (14.37) | 34.06 (13.00) |
| Number of gambling activities (past year) | 2.49 (1.79) | 5.54 (3.19) | 6.35 (3.25) |
| Most units drank in one day (past week frame) | 5.41 (6.72) | 8.58 (9.42) | 12.3 (17.7) |
| General Health | 1.96 (0.88) | 1.86 (0.78) | 2.27 (0.97) |
| Age of first gamble | 19.21 (10.68) | 16.63 (6.18) | 16.19 (6.16) |
| Estimated monthly gambling spend | 18.44 (58.3) | 109.72 (242.21) | 288.28 (518.19) |
| Sex (REF: Male) | 0.489 | 0.776 | 0.817 |
| *Marital Status:* |  |  |  |
| Married | 0.637 | 0.444 | 0.417 |
| Separated/Divorced | 0.084 | 0.06 | 0.099 |
| Single | 0.216 | 0.479 | 0.484 |
| Widowed | 0.064 | 0.017 | 0 |
| Smoker (REF: Yes) | 0.262 | 0.43 | 0.566 |
| Drinker (REF: Yes) | 0.78 | 0.817 | 0.712 |
| *Proportion played:* |  |  |  |
| National Lottery | 0.804 | 0.802 | 0.791 |
| Scratchcard | 0.321 | 0.549 | 0.636 |
| Other Lottery | 0.342 | 0.36 | 0.39 |
| Bingo (Online + Land) | 0.114 | 0.22 | 0.2 |
| Pools | 0.05 | 0.235 | 0.257 |
| Slot Machines | 0.155 | 0.524 | 0.564 |
| EGM at bookmaker | 0.039 | 0.362 | 0.505 |
| Poker | 0.018 | 0.197 | 0.137 |
| Casino games (Online + Land) | 0.058 | 0.327 | 0.388 |
| Online fruit/slot machine/ instant win | 0.029 | 0.192 | 0.27 |
| Horse racing | 0.212 | 0.381 | 0.477 |
| Dog racing | 0.052 | 0.186 | 0.307 |
| Spread betting | 0.009 | 0.083 | 0.14 |
| Private betting | 0.14 | 0.45 | 0.459 |
| Other sports bets | 0.1 | 0.443 | 0.495 |
| Other bets | 0.046 | 0.231 | 0.335 |
| Bingo in person | 0.1 | 0.166 | 0.176 |
| Bingo online | 0.021 | 0.081 | 0.064 |
| Slots (inc FOBT prompt) | 0.157 | 0.542 | 0.564 |
| FOBT | 0.033 | 0.322 | 0.378 |
| Casino person | 0.043 | 0.216 | 0.3 |
| Casino online | 0.02 | 0.178 | 0.176 |
| Online gaming | 0.056 | 0.301 | 0.399 |
| Horse in person | 0.194 | 0.346 | 0.453 |
| Horse online | 0.025 | 0.081 | 0.067 |
| Dogs in person | 0.05 | 0.159 | 0.283 |
| Dogs online | 0.002 | 0.035 | 0.036 |
| Sports/other betting land | 0.1 | 0.421 | 0.534 |
| Other betting in person | 0.009 | 0.007 | 0.085 |
| Sports online | 0.026 | 0.17 | 0.083 |
| Sports in person | 0.081 | 0.37 | 0.478 |
| Online betting exchange | 0.01 | 0.061 | 0.042 |
| Online bookmaker | 0.034 | 0.177 | 0.136 |
| Online bookmaker + exchange | 0.04 | 0.197 | 0.155 |
| Online gaming + lottery | 0.164 | 0.394 | 0.408 |
| Online gaming | 0.06 | 0.343 | 0.399 |

Please see note below

Note for Tables S24 and S25:

‘Poker’ specifically refers to poker games played for money at a league, pub, tournament or club.

‘FOBT’ refers to a classification (B2) of gaming machines in the United Kingdom. These are rapid play machines with a maximum stake of £100 (although must individually enter £10 notes into the machine), and a maximum payout of £500. The rate of return to player is approximately 97%. The games on these machines are often presented in the form of casino style games (e.g. roulette, poker), but with a fixed odds of success determined by the machine. These are similar to electronic gaming and poker machines elsewhere in the world.

‘Online gaming’ in the BGPS 2010 refers to engagement in slot machine/instant win style games, casino games, online bingo and online pools.

References to ‘land’ forms of gambling capture the distinction between online and ‘land-based’ gambling (i.e. gambling on the premises of a bookmaker, casino, racecourse etc.) in British gambling legislation.

Table S26 Demographic and gambling behaviour variables for each latent class for the HSE and SHS 2012 DSM > 0 LCA.

|  | Class 1 (*S.D.*) | Class 2 (*S.D*) | Class 3 (*S.D.*) |
| --- | --- | --- | --- |
| Age | 46.66 (17.49) | 36.02 (16.14) | 39.09 (17.1) |
| Number of gambling activities (past year) | 2.11 (1.5) | 4.62 (3.38) | 5.42 (4.25) |
| Units drank in previous week | 12.86 (21.98) | 17.47 (23.64) | 15.89 (27.98) |
| Most units drank in one day (past week) | 4.48 (5.86) | 7.54 (9.21) | 5.86 (8.99) |
| General health | 1.93 (0.895) | 1.95 (0.915) | 2.12 (1.05) |
| GHQ Score | 1.34 (2.55) | 1.88 (2.88) | 2.99 (3.63) |
| Sex (REF: Male) | 0.5 | 0.731 | 0.786 |
| *Marital Status:* |  |  |  |
| Married | 0.662 | 0.461 | 0.443 |
| Separated/Divorced | 0.087 | 0.074 | 0.071 |
| Single | 0.203 | 0.452 | 0.444 |
| Widowed | 0.049 | 0.012 | 0.042 |
| Previously smoked (REF: Y) | 0.592 | 0.614 | 0.629 |
| Current smoker (REF: Y) | 0.211 | 0.319 | 0.298 |
| Current drinker (REF: Y) | 0.846 | 0.834 | 0.676 |
| Proportion of class members played: |  |  |  |
| National Lottery | 0.824 | 0.776 | 0.773 |
| Scratchcards | 0.295 | 0.531 | 0.449 |
| Other Lottery | 0.225 | 0.233 | 0.398 |
| Pools | 0.032 | 0.203 | 0.214 |
| Bingo | 0.083 | 0.105 | 0.238 |
| Slot Machines | 0.1 | 0.34 | 0.255 |
| FOBT in bookmaker | 0.028 | 0.303 | 0.32 |
| Casino games | 0.041 | 0.204 | 0.223 |
| Poker | 0.012 | 0.111 | 0.202 |
| Online gaming | 0.034 | 0.273 | 0.242 |
| Online betting | 0.067 | 0.269 | 0.311 |
| Betting exchange | 0.01 | 0.065 | 0.13 |
| Horse racing | 0.154 | 0.324 | 0.371 |
| Dog racing | 0.04 | 0.117 | 0.15 |
| Sports betting | 0.057 | 0.311 | 0.461 |
| Other betting | 0.01 | 0.102 | 0.156 |
| Spread betting | 0.004 | 0.053 | 0.111 |
| Private betting | 0.075 | 0.241 | 0.183 |
| Other gambling | 0.019 | 0.063 | 0.232 |

FIGURE LEGENDS

Figure S1. Plot of response probabilities for each item of the DSM-IV Pathological Gambling derived assessment items, three latent class solutions. Latent classes are sorted by severity/group membership (largest first).
